# Anti-CELA1 antibody KF4 prevents emphysema by inhibiting stretch-mediated remodeling

**DOI:** 10.1172/jci.insight.169189

**Published:** 2024-01-09

**Authors:** Mohit Ojha, Noah J. Smith, Andrew J. Devine, Rashika Joshi, Emily M. Goodman, Qiang Fan, Richard Schuman, Aleksey Porollo, J. Michael Wells, Ekta Tiwary, Matthew R. Batie, Jerilyn Gray, Hitesh Deshmukh, Michael T. Borchers, Samuel A. Ammerman, Brian M. Varisco

**Affiliations:** 1 Lincoln Medical Center and Mental Health Center, New York, New York, USA.; 2 College of Medicine, University of Cincinnati, Cincinnati, Ohio, USA.; 3 Heritage College of Osteopathic Medicine, Ohio University, Athens Ohio, USA.; 4 Critical Care Medicine, Cincinnati Children’s Hospital Medical Center, Cincinnati, Ohio, USA.; 5 College of Medicine, University of Arkansas for Medical Sciences, Little Rock, Arkansas, USA.; 6 Antibody and Immunoassay Consultants, Rockville, Maryland, USA.; 7 Center for Autoimmune Genomics and Etiology, Cincinnati Children’s Hospital Medical Center, Cincinnati, Ohio, USA.; 8 University of Alabama at Birmingham School of Medicine, Birmingham, Alabama, USA.; 9 UAB Lung Health Center, Birmingham, Alabama, USA.; 10 Clinical Engineering, and; 11 Perinatal Institute, Center for Perinatal Immunity, Cincinnati Children’s Hospital Medical Center, Cincinnati, Ohio, USA.; 12 Division of Pulmonary and Critical Care Medicine, University of Cincinnati, Cincinnati, Ohio, USA.; 13 School of Medicine, The Ohio State University, Columbus, Ohio, USA.; 14 Arkansas Children’s Research Institute, Little Rock, Arkansas, USA.

**Keywords:** Aging, Pulmonology, Extracellular matrix, Respiration

## Abstract

There are no therapies to prevent emphysema progression. Chymotrypsin-like elastase 1 (*CELA1*) is a serine protease that binds and cleaves lung elastin in a stretch-dependent manner and is required for emphysema in a murine antisense oligonucleotide model of α-1 antitrypsin (AAT) deficiency. This study tested whether *CELA1* is important in strain-mediated lung matrix destruction in non–AAT-deficient emphysema and the efficacy of CELA1 neutralization. Airspace simplification was quantified after administration of tracheal porcine pancreatic elastase (PPE), after 8 months of cigarette smoke (CS) exposure, and in aging. In all 3 models, *Cela1^–/–^* mice had less emphysema and preserved lung elastin despite increased lung immune cells. A CELA1-neutralizing antibody was developed (KF4), and it inhibited stretch-inducible lung elastase in ex vivo mouse and human lung and immunoprecipitated CELA1 from human lung. In mice, systemically administered KF4 penetrated lung tissue in a dose-dependent manner and 5 mg/kg weekly prevented emphysema in the PPE model with both pre- and postinjury initiation and in the CS model. KF4 did not increase lung immune cells. CELA1-mediated lung matrix remodeling in response to strain is an important contributor to postnatal airspace simplification, and we believe that KF4 could be developed as a lung matrix–stabilizing therapy in emphysema.

## Abstract 2

CELA1 mediates emphysema progression in multiple models and is responsible for strain-induced lung elastase activity.

## Introduction

The lung is a complex and delicate organ, with a gas-exchange surface area of approximately 150 m^2^. This surface area is accommodated by the thoracic cavity via a highly stereotyped pattern of branching airways terminating in thin alveolar sacs that are septated and perfused by an extensive capillary network. Emphysema is the term used for acquired alveolar simplification that results in reduced gas exchange capacity. Alveolar simplification is an element of many respiratory diseases such as connective tissue disorders, α-1 antitrypsin (AAT) deficiency, chronic obstructive pulmonary disease (COPD), and aging. COPD accounts for approximately $50 billion in US health care expenditures annually, and before the COVID-19 pandemic, COPD was the third leading cause of death worldwide (1). While a host of immune, epithelial, stromal, and vascular cell defects have been described, an important defect in COPD is loss of lung matrix (2). This can be observed quite strikingly in comparing lung computed tomography images of healthy and COPD patients, in which the latter have loss of large areas of matrix and associated cells (3).

Among the host of extracellular matrix components in the lung, none is more important that elastin. Elastin fibers are composed of many microfibril-associated proteins and a core of hydrophobic tropoelastin monomers and arranged in a repetitive overlapping pattern (4). When tension is applied to elastin fibers, potential energy is stored as these hydrophobic domains are separated, giving the lung its elasticity, which is key to passive exhalation. While many proteases degrade lung elastin fibers, we have previously described a unique role for chymotrypsin-like elastase 1 (*CELA1*) (5). CELA1 is a serine protease initially described as a digestive enzyme but was later shown to have developmentally regulated expression pattern in alveolar type 2 (AT2) cells (6, 7). In the lung, CELA1 mRNA and protein are increased with strain (8), and like other pancreatic elastases, CELA1 binds to hydrophobic domains of tropoelastin when exposed by stretch (8). The normal role for CELA1 is to reduce postnatal elastance, and *Cela1^–/–^* mice are phenotypically normal except for having higher lung elastance than wild-type (WT) mice (5). CELA1 is neutralized by AAT and is required for development of emphysema in an antisense oligonucleotide mouse model of AAT-deficient emphysema (6).

There are multiple conceptual models of emphysema pathobiology. Perhaps the oldest is the altered protease/antiprotease balance model developed after the discovery of AAT by Laurell and Erikson in 1964 (9). It holds that unopposed protease activity leads to tissue destruction and emphysema. The accelerated aging model is based on similarities in airspace architecture between COPD and aged human lung and similar changes in cellular senescence, oxidative stress, and matrix architecture (10). The biomechanical model holds that strain is distributed over airspace walls and associated matrix structures and that alveolar wall disruption increases strain on adjacent walls and predisposes them to failure. This model has been validated in computational and animal models (11–14). We hypothesized that the stretch-dependent remodeling activity of CELA1 mechanistically unites these 3 models and promotes feed-forward alveolar wall destruction that can be prevented using a neutralizing antibody.

## Results

### Mouse models of lung injury and emphysema have increased Cela1 expression.

**Figure 1:**
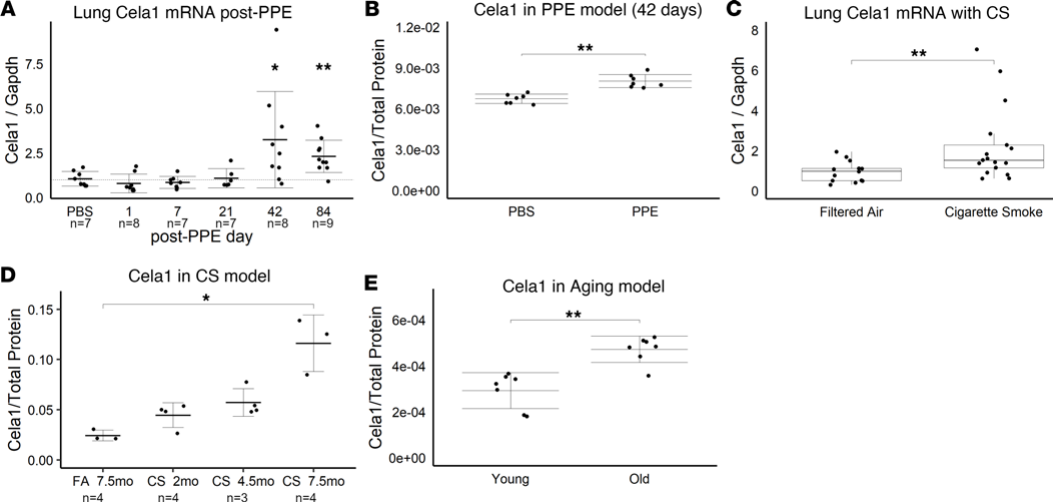
Cela1 expression in 3 mouse models of emphysema. (**A**) Following tracheal administration of 2 units porcine pancreatic elastase (PPE) or PBS, normalized lung *Cela1* mRNA levels were elevated at 42 and 84 days. *P* < 0.01 by 1-way ANOVA and Holm-Šidák post hoc comparisons are shown by *P < 0.05 and **P < 0.01 compared with PBS control. Number of mice per group is indicated by *n*. (**B**) Western blot of 42-day post-PPE lung homogenate, which had more native Cela1 protein than PBS-treated lung. **P < 0.01 by 2-tailed Welch’s *t* test. *n* = 7 per group. (**C**) Compared with filtered-air controls, the lungs of mice exposed to whole-body cigarette smoke (CS) for 8 months had increased normalized *Cela1* mRNA levels. **P < 0.01 by Mann-Whitney *U* test. (**D**) With CS exposure, lung native Cela1 protein was increased. *P* < 0.05 by 1-way ANOVA and Holm-Šidák post hoc comparisons are shown by *P < 0.05. FA, filtered air. (**E**) The lungs of 72- to 75-week-old mice had more native Cela1 protein than those of mice at the age of 8–10 weeks. *n* = 7 per group. **P < 0.01 by 2-tailed Welch’s *t* test.

We quantified *Cela1* lung mRNA and protein levels in 3 different mouse models of lung injury. Following PPE administration, lung *Cela1* mRNA and native Cela1 protein levels began increasing on day 21 (Figure 1, A and B), but AAT-neutralized Cela1 levels were not different (Supplemental Figure 1; supplemental material available online with this article; https://doi.org/10.1172/jci.insight.169189DS1). In the cigarette smoke (CS) exposure model, *Cela1* mRNA levels were almost doubled after 8 months (Figure 1C). Native and AAT-neutralized protein levels were slightly elevated after 2 and 4.5 months of exposure, but significantly elevated after 7.5 months (Figure 1D and Supplemental Figure 1). In the aging model, there was no difference in mRNA levels, but native Cela1 protein levels were increased in aged lung (Figure 1E and Supplemental Figure 1). Although there were differences between models, there was a pattern of increased *Cela1* expression in each mouse model of emphysema.

### Cela1-deficient mice have preserved alveolar architecture in multiple models of emphysema.

**Figure 2:**
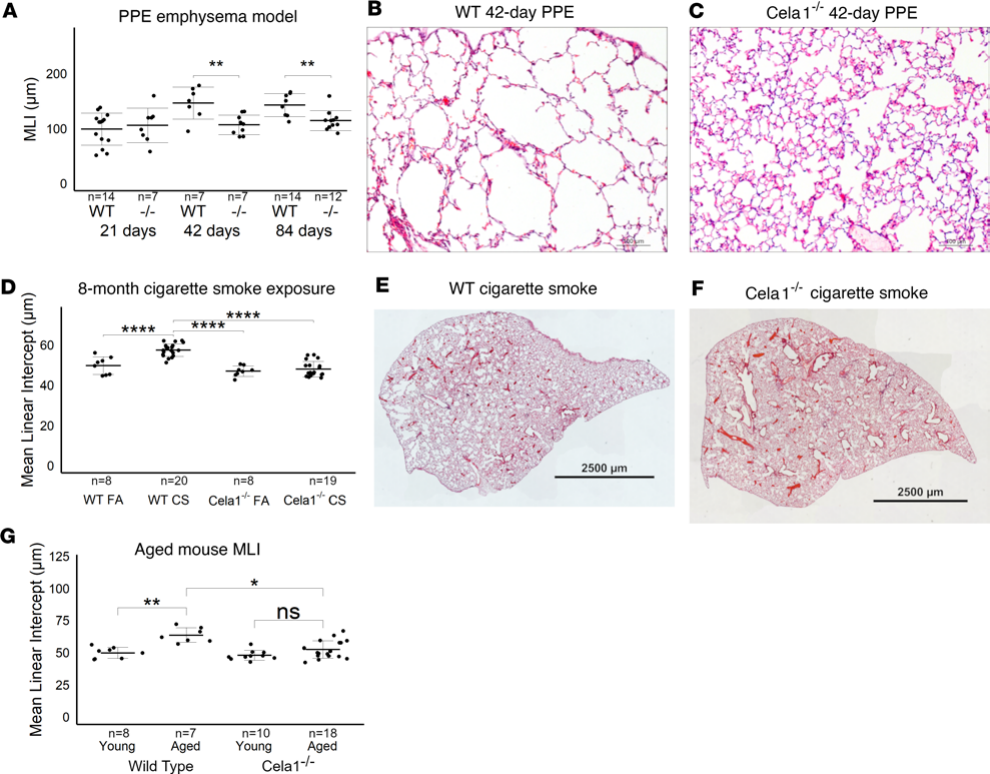
Protection of *Cela1^–/–^* mice in 3 emphysema models. (**A**) At 21 days after airway administration of porcine pancreatic elastase (PPE), *Cela1^–/–^* and WT mice had a similar level of emphysema. However, at 42 and 84 days, WT mice experienced progression of emphysema as evidenced by greater mean linear intercept (MLI) values, while *Cela1^–/–^* mice did not. *P* < 0.001 by 1-way ANOVA and Holm-Šidák post hoc comparisons are shown by **P < 0.01. Dashed line represents average MLI of WT PBS-treated mice. *n* is the number of mice per group. (**B**) Representative 42-day photomicrographs of WT and (**C**) *Cela1^–/–^* lungs are shown. Scale bars: 100 μm. (**D**) *Cela1^–/–^* mice exposed to 4 hours of cigarette smoke 5 days per week for 8 months had almost no detectable airspace simplification, while such emphysema was detected in WT mice. FA, filtered air. *P* < 0.0001 by 1-way ANOVA and Holm-Šidák post hoc comparisons are shown by ****P < 0.0001. (**E**) Representative tile-scanned images of middle lobes of WT and (**F**) *Cela1^–/–^* mice are shown. Scale bars: 2,500 μm. (**G**) In comparing lung images of untreated mice aged 72 to 75 weeks, the MLI of WT mice was significantly increased, while that of *Cela1^–/–^* mice was not significantly larger (NS = not significant) and aged *Cela1^–/–^* mice had less age-related alveolar simplification than WT. *P* < 0.001 by 1-way ANOVA and Holm-Šidák post hoc comparisons are shown by ***P < 0.001.

To test whether *CELA1* has a general role in airspace simplification, we tested *Cela1*-deficient mice in these models. *Cela1^–/–^* mice were not protected from initial injury and airspace destruction 21 days after administration of tracheal PPE, but unlike WT mice, they did not demonstrate emphysema progression at 42 and 84 days (Figure 2, A–C). Following 8 months of CS exposure, *Cela1^–/–^* mice had less emphysema than WT mice (Figure 2, D–F), and they also had less age-related alveolar simplification (Figure 2G). Although we previously reported that Cela1 mRNA and protein were increased in the mouse partial pneumonectomy model of lung regeneration (8), we found no difference in compensatory lung regrowth at 7 or 28 days (Supplemental Figure 2A). In colony-forming-efficiency experiments, there was a trend toward increased colony-forming efficiency in *Cela1^–/–^* mice 21 days after PPE (*P* = 0.1, ~50% increase compared with all other groups). The efficiencies of WT PBS-, WT PPE-, and *Cela1^–/–^* PBS-treated mice were similar (Supplemental Figure 2B). There was no difference by sex in any of the emphysema or compensatory lung growth experiments (Supplemental Figures 2–4). Taken together, these data show a central role for *CELA1* in progressive alveolar destruction but not in protection from initial injury but cannot exclude some element of enhanced repair in *Cela1^–/–^* mice.

### Cela1-deficient mice have less elastin degradation and senescence than WT mice.

**Figure 3:**
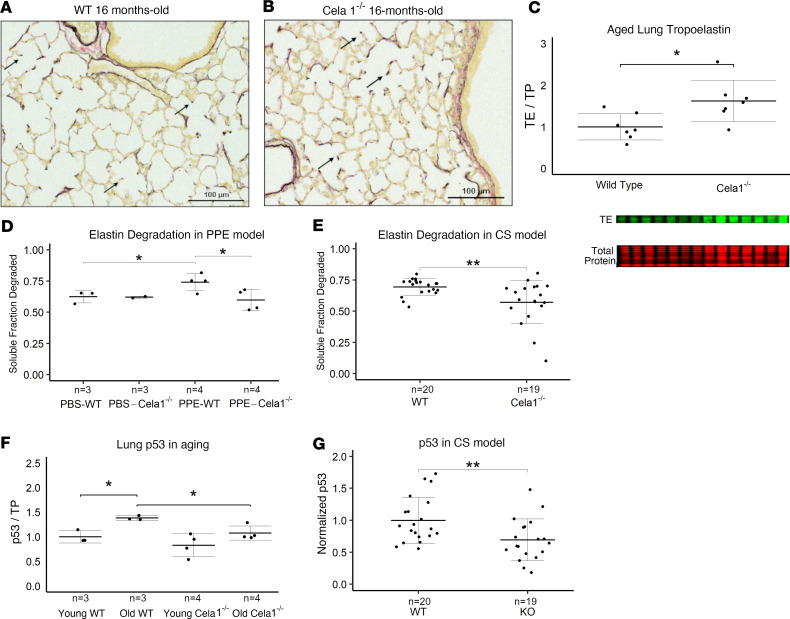
Lung elastin degradation and senescence in 3 mouse emphysema models. (**A**) Photomicrographs of Hart-stained lung sections of aged WT and (**B**) *Cela1^–/–^* mouse lung show both preservation of alveolar structure in *Cela1^–/–^* mice and preservation of alveolar septal tip elastin bands (arrows). Scale bars: 100 μm. (**C**) By Western blot, the lungs of *Cela1^–/–^* mice have a greater amount of total lung tropoelastin (TE). Normalization is by total protein (TP) and comparison is by 2-tailed Welch’s *t* test. *P < 0.05. *n* = 7 per group. (**D**) At 42 days after PPE, the lungs of WT mice had more degraded elastin than PBS-treated WT mice and PPE-treated *Cela1^–/–^* mice. *P* < 0.05 by 1-way ANOVA and Holm-Šidák post hoc comparisons are shown by *P < 0.05. Number of specimens per group is noted by *n*. (**E**) After 8 months of exposure, the lungs of cigarette smoke–exposed (CS-exposed) *Cela1^–/–^* mice had less degraded elastin than the lungs of WT mice. (**F**) p53 levels were increased in aged WT but not *Cela1^–/–^* lungs. *P < 0.05 by 2-tailed Welch’s *t* test. (**G**) The lungs of CS-exposed *Cela1^–/–^* mice had less p53 than the lungs of WT mice. **P < 0.01 by 2-tailed Welch’s *t* test.

Both senescence (10, 15) and matrix degradation (16, 17) are associated with emphysema. We noticed that the elastin architecture of aged *Cela1^–/–^* mouse lung appeared more intact than that of aged WT mice (Figure 3, A and B). Western blotting demonstrated more intact tropoelastin in knockout lungs (Figure 3C). At 42 days after PPE, *Cela1^–/–^* mice had less degraded elastin than WT (Figure 3D and Supplemental Figure 3), consistent with Cela1-mediated matrix destruction at this time point. Similarly, *Cela1^–/–^* mice had less degraded elastin after 8 months of CS exposure (Figure 3E and Supplemental Figure 4). Even though CS-treated mice had expected changes in lung collagen after CS exposure, there were no difference in levels of the collagen matrikines proline-glycine-proline (PGP) or acyl-PGP (Supplemental Figure 5). In contrast, the senescence marker p53 was higher in aged WT lungs compared with aged *Cela1^–/–^* and young WT (Figure 3F) and was higher in WT compared with *Cela1^–/–^* lungs after CS exposure (Figure 3G and Supplemental Figure 5). Taken together, these data show that *Cela1*-deficient mice have attenuation of at least 2 processes implicated in emphysema progression.

### Cela1^–/–^ mice have more lung immune cells than WT mice in multiple models.

**Figure 4:**
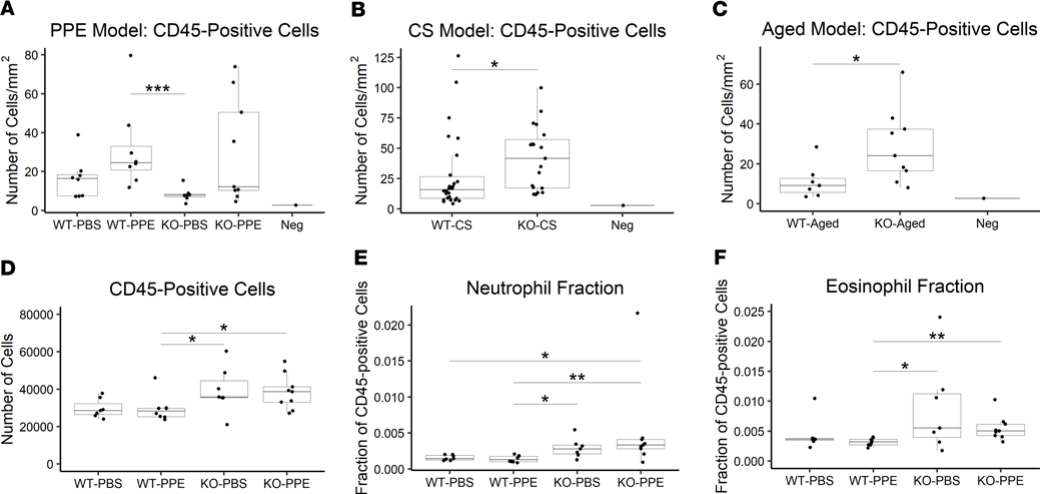
Immune cells in *Cela1*-deficient mice. (**A**) In lung sections from WT and *Cela1^–/–^* (KO) mice treated with tracheal PBS or porcine pancreatic elastase (PPE, *n* = 8 for all groups) immunostained for the leukocyte marker CD45, the only notable difference was fewer immune cells in KO-PBS mice compared with WT-PPE. Neg is the number of cells identified with secondary antibody staining alone. (**B**) After 8 months of cigarette smoke exposure, the number of leukocytes was increased in KO mice (*n* = 19) compared with WT (*n* = 20). (**C**) Similarly, in 72- to 75-week-old mouse lungs, more immune cells were present in KO (*n* = 9) compared with WT (*n* = 7). (**D**) In a different experiment, we used flow cytometry to quantify immune cell populations. Two hundred and fifty thousand cells were analyzed for each mouse. *n* = 7, 8, 8, and 9 for WT-PBS, WT-PPE, KO-PBS, and KO-PPE, respectively. More CD45-positive cells were present in KO lungs. (**E** and **F**) In analysis of immune cell populations, only the fractional increases in neutrophils and eosinophils were notable. For all analyses, *P* < 0.05 by Kruskal-Wallis test and *P < 0.05, **P < 0.01, ***P < 0.001 by Dunn’s post hoc test.

As inflammation plays a key role in emphysema pathogenesis, we evaluated lung immune cell composition using 2 different methods. First, we quantified the number of CD45-positive cells in the lungs of previously evaluated mice using automated quantitative image analysis of immunohistochemically stained lungs sections. At 42 days, PPE showed a trend toward increased numbers of CD45-positive cells, but there was no clear difference between WT and *Cela1^–/–^* lungs (Figure 4A). After 6 months of CS exposure, *Cela1^–/–^* mice had more CD45-positive cells (Figure 4B), and the same was true in the lungs of 15-month-old mice (Figure 4C). Flow cytometry from the lungs of mice treated with PBS or PPE and evaluated at 21 days showed 30% more CD45-positive cells in the lungs of *Cela1^–/–^* mice compared with WT (Figure 4D), with the greatest differences being in the fractional abundance of neutrophils and eosinophils (Figure 4, E and F), but there were very few differences between PBS and PPE in either group (Supplemental Figure 6). These neutrophil data are in contrast with lower lung myeloperoxidase activity levels in AAT-deficient and *Cela1*-deficient mice compared with AAT-deficient after 6 months of CS exposure (18). The weight of evidence indicates that *Cela1^–/–^* mice have more lung immune cells than WT at baseline, but the significance of this finding is unclear.

### CELA1-binding and stretch-inducible elastase activity in human lung.

The demographic characteristics of the participants from whom control, smoker, and COPD specimens were obtained for this study are presented in Supplemental Table 1.

**Figure 5:**
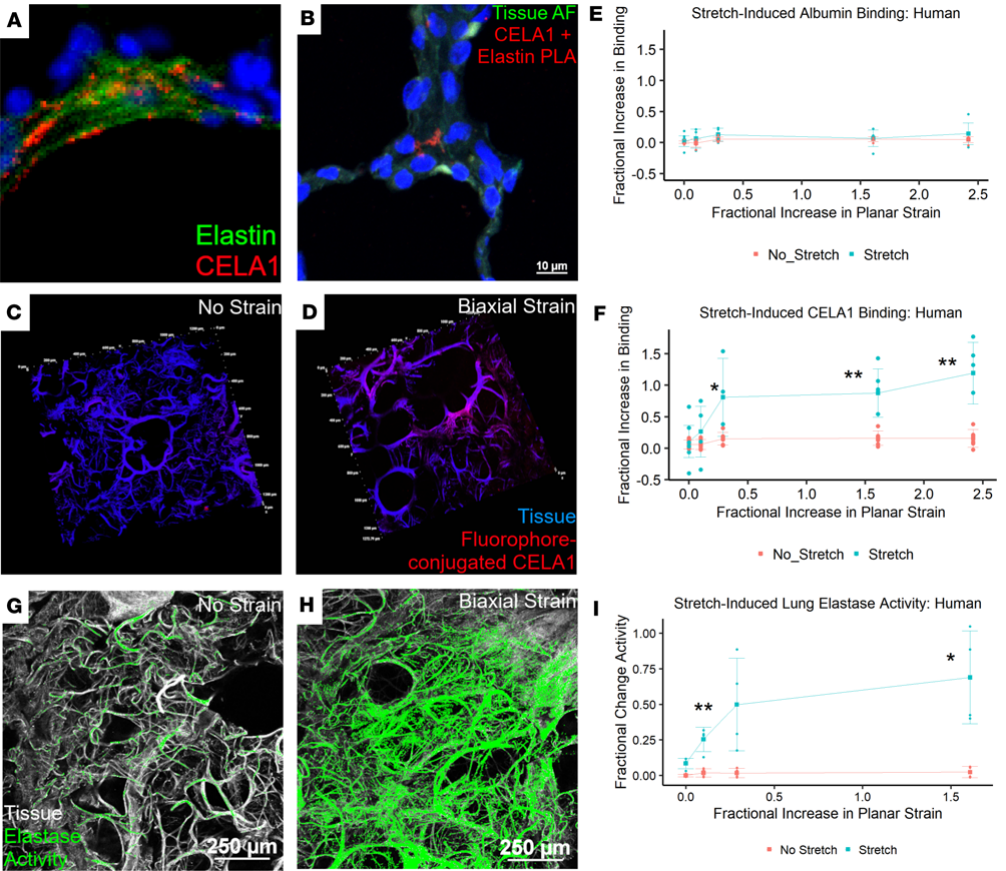
CELA1 in human lung and stretch-dependent binding and elastase activity. (**A**) Immunofluorescence imaging for human tropoelastin (green) and CELA1 (red) demonstrated CELA1 protein found near elastin fibers in the airspace walls. Scale bar: 100 μm. (**B**) Proximity ligation assay (PLA) was performed in human lung using anti-tropoelastin and anti-CELA1 antibodies. Red signal is obtained when antibodies are located within 40 nm of each other. This red signal was observed in the alveolar interstitium. (**C**) Using fluorophore-conjugated CELA1, human lung tissue sections demonstrated little binding of CELA1 (red) to lung tissue (blue) in the absence of strain. Tissue section is 100 μm thick and each tick mark represents 200 μm. Dashed line highlights a comparison region. (**D**) When subjected to biaxial strain, CELA1 binding to lung tissue increased. The dashed line highlights the same region as the prior panel. (**E**) Fluorophore-conjugated albumin binding to lung tissue did not increase with strain. This channel was omitted from images for clarity. (**F**) Quantification of CELA1 binding in 4 independent lung specimens shows that binding increases with increasing levels of strain to approximately 5-fold. *P < 0.05, **P < 0.01 by 2-tailed Welch’s *t* test. (**G**) Using a fluorophore-conjugated and quenched soluble elastin substrate for in situ zymography, human lung tissue (white) does not have appreciable elastase activity (green) at baseline. Scale bars: 250 μm. (**H**) When subjected to biaxial strain, there is an increase in lung elastase activity. (**I**) Lung elastase activity increases with strain by approximately 8-fold. *P < 0.05, **P < 0.01 by 2-tailed Welch’s *t* test.

In human lung, CELA1 was present in the extracellular matrix in association with elastin fibers (Figure 5A), with confirmed separation of less than 20 nm by proximity ligation assay (Figure 5B). CELA1 binds and cleaves non–cross-linked tropoelastin at hydrophobic domains, and we hypothesized that like other pancreatic elastases (19), CELA1 binds and cleaves tropoelastin when hydrophobic domains are exposed by strain. In mouse lung, strain-increased CELA1 binding to lung elastin increased approximately 4-fold (8). In human lung, we quantified the binding of fluorophore-conjugated albumin, fluorophore-conjugated CELA1, and tissue elastolytic activity by fluorescent elastin in situ zymography. The independent variable in these experiments was fractional increase in planar strain, which is the change in silicone mount imaging area, as previously reported (6). In human lung, with increasing levels of biaxial strain the binding of fluorophore-conjugated CELA1 but not albumin increased (Figure 5, C–F, and Supplemental Figure 7). Biaxial stretch induces lung elastase activity in WT but not *Cela1^–/–^* mouse lung sections (6). In human lung sections, biaxial strain also increased lung elastase activity (Figure 5, G–I), suggesting that similar strain-related remodeling programs are operative in human and mouse lung.

### Human lung CELA1 mRNA levels correlate with elastolytic activity.

**Figure 6:**
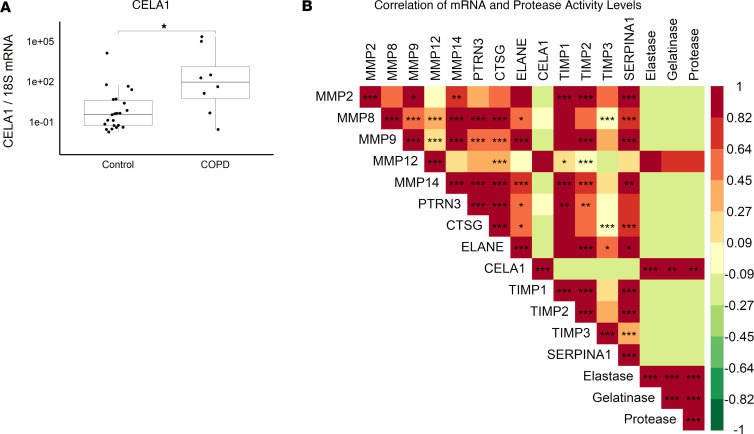
CELA1, other proteases, and human lung elastase activity. (**A**) In 23 control and 8 COPD lung homogenates, *CELA1* mRNA levels were variable but overall higher in COPD lungs than smoker controls but not different from nonsmoker control. *P < 0.05 by Mann-Whitney *U* test. (**B**) mRNA levels of matrix metalloproteinase 2 (*MMP2*), *MMP8*, *MMP9*, *MMP12*, *MMP14,* proteinase-3 (*PTRN3*), cathepsin G (*CTSG*), neutrophil elastase (*ELANE*), *CELA1*, tissue inhibitor of metalloproteinase-1 (*TIMP1*), *TIMP2*, *TIMP3*, and α1-antitrypsin (*SERPINA1*) as well as tissue protease, gelatinase, and elastase activities were compared by Spearman’s rank correlation with Bonferroni’s correction for multiple comparisons. *CELA1* and *MMP12* mRNA levels correlated with each other and with tissue protease, elastase, and gelatinase levels. The other proteases and antiproteases were generally correlated with each other. *P < 0.05, **P < 0.01, ***P < 0.001.

Since most proteases reported to have a role in emphysema are expressed by leukocytes and CELA1 is expressed in AT2 cells, we wondered whether there might be distinct remodeling programs that could be identified in human lung. Based on the premise that expression of genes in different programs would be co-regulated, we quantified CELA1 mRNA and protease activity in lung homogenates from 23 non–lung organ donors and 8 individuals with COPD. *CELA1* mRNA levels varied logarithmically but were higher in COPD with emphysema specimens compared with control (Figure 6A). Similar patterns were observed with matrix metalloproteinase-8 (*MMP8*), *MMP9*, *MMP12*, *MMP14*, proteinase-3 (*PRTN3*), cathepsin G (*CTSG*), neutrophil elastase (*ELANE*), tissue inhibitor of matrix proteinase-1 (*TIMP1*), *TIMP2*, *TIMP1*, and AAT (*SERPINA1*), but not *MMP2* (Supplemental Figure 8). In comparing gene mRNA levels with protease, elastase, and gelatinase activities, the mRNA levels of most proteases and antiproteases positively correlated with each other but not with *CELA1*. Among the assayed proteases, only *CELA1* mRNA levels positively and significantly correlated with lung elastase and protease activity levels (Figure 6B). These data suggest that CELA1 is part of a different remodeling program than the majority of proteases known to be important in the development of emphysema.

### CELA1-expressing cells cluster in COPD lung.

**Figure 7:**
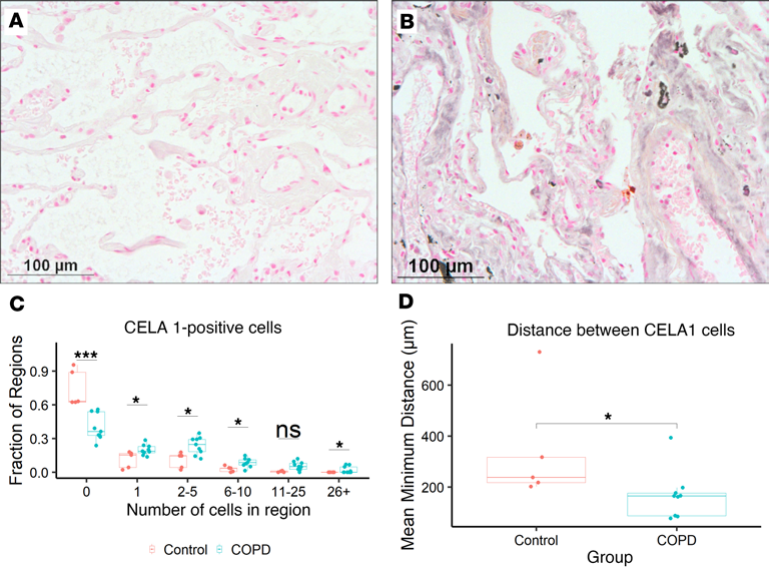
Distribution of CELA1-expressing cells in COPD. (**A**) In control lung, CELA1-expressing cells were rare. Representative ×20 photomicrograph shown. (**B**) In COPD lung sections, CELA1-expressing cells were more numerous. Scale bars: 100 μm. (**C**) Five control and 9 COPD specimens were stained for CELA1 and tile scanned to assess the distribution of CELA1-expressing cells in COPD. Images were overlaid with 1 mm × 1 mm regions of interest and the number of cells in each region counted. COPD specimens had a greater number of grids with CELA1-positive cells. *P < 0.05, ***P < 0.001 by Mann-Whitney *U* test. (**D**) The average minimum distance between each CELA1-positive cell and its nearest neighbor was determined for each section. CELA1-positive cells were closer together in COPD specimens. Mann-Whitney *U* test *P* value shown on the plot.

By immunohistochemical staining and analysis, COPD lung sections had greater abundance of CELA1-positive cells (Figure 7, A–D), but the distribution of these cells were inhomogeneous. To quantify this observation, we used tile-scanned images of COPD and control lung to quantify the number of CELA1-positive cells within each region of a 1-mm square grid and also measured the distance from each CELA1-positive cell to its nearest CELA1-positive neighbor. While the majority of 1-mm^2^ regions had zero or 1 CELA1-positive cell, there were more fields with 2 or more CELA1-positive cells in COPD lung compared with control (Figure 7C). The average distance between CELA1-positive cells was less in COPD lung (Figure 7D). Though limited by the 2-dimensional nature of this analysis, these data show that CELA1 expression in COPD lung is both increased and inhomogeneous. When considered in the context of increased binding of CELA1 to lung elastin with strain (Figure 5), the data suggest that more CELA1 is poised for elastin remodeling given a permissive biomechanical environment.

### CELA1 genomics.

The gene *CELA1* was not previously identified in COPD genome-wide association studies (GWAS) (20). *CELA1* is highly conserved in placental mammals (6) and *CELA1* mutations in humans are rare, with the most common predicted loss-of-function mutation having an allele frequency of 0.03% (Table 1) (21–23), and no predicted gain-of-function mutations are known (24, 25). As homozygous loss of function would be protective and occur in 1:11.7 million individuals, COPD GWAS would have been underpowered to identify an association of the *CELA1* locus with disease.

### Development of the KF4 anti-CELA1 antibody and inhibition of stretch-inducible elastase activity.

**Figure 8:**
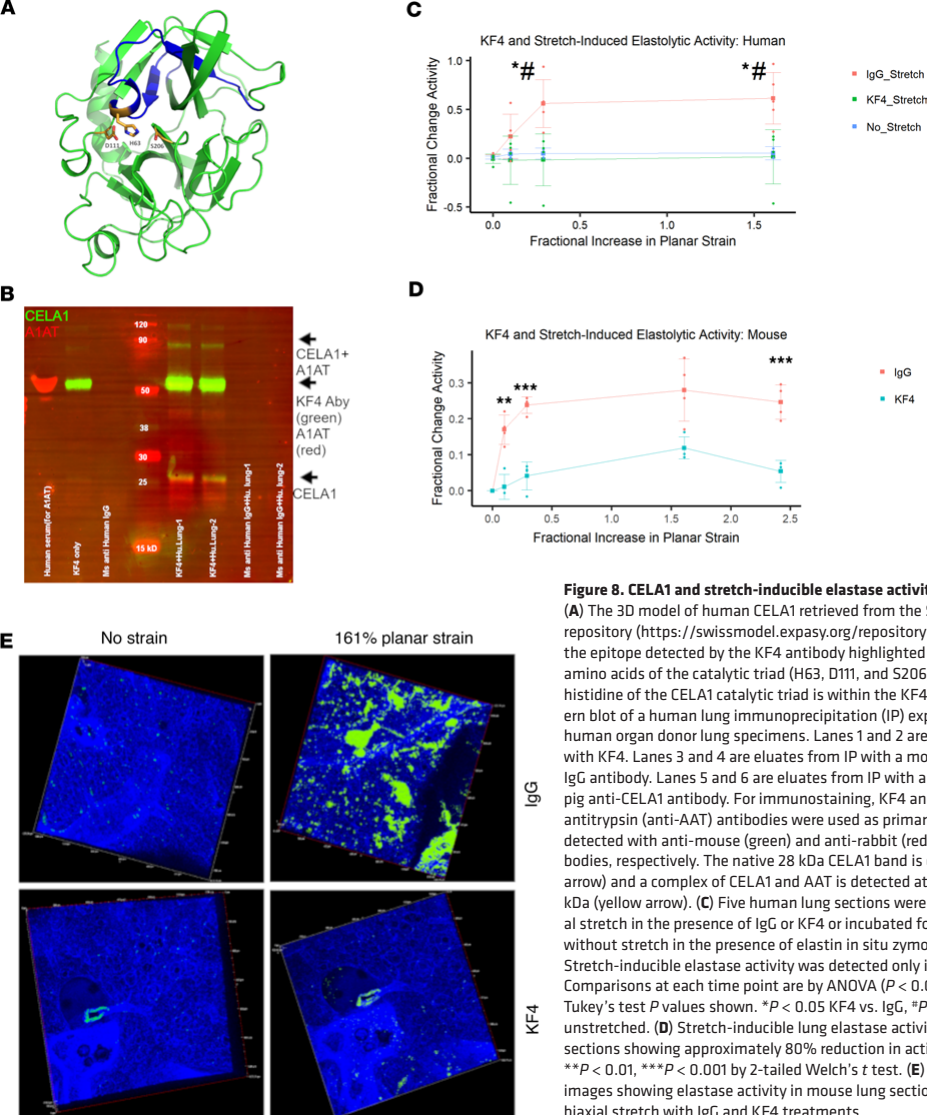
CELA1 and stretch-inducible elastase activity in human lung. (**A**) The 3D model of human CELA1 retrieved from the Swiss-Model repository (https://swissmodel.expasy.org/repository; ID: Q9UNI1) with the epitope detected by the KF4 antibody highlighted in blue. The 3 amino acids of the catalytic triad (H63, D111, and S206) are shown. The histidine of the CELA1 catalytic triad is within the KF4 epitope. (**B**) Western blot of a human lung immunoprecipitation (IP) experiment using 2 human organ donor lung specimens. Lanes 1 and 2 are eluates from IP with KF4. Lanes 3 and 4 are eluates from IP with a mouse anti–human IgG antibody. Lanes 5 and 6 are eluates from IP with a polyclonal guinea pig anti-CELA1 antibody. For immunostaining, KF4 and anti–human α-1 antitrypsin (anti-AAT) antibodies were used as primary antibodies and detected with anti-mouse (green) and anti-rabbit (red) secondary antibodies, respectively. The native 28 kDa CELA1 band is detected (green arrow) and a complex of CELA1 and AAT is detected at approximately 60 kDa (yellow arrow). (**C**) Five human lung sections were subjected to biaxial stretch in the presence of IgG or KF4 or incubated for equivalent times without stretch in the presence of elastin in situ zymography substrate. Stretch-inducible elastase activity was detected only in the IgG group. Comparisons at each time point are by ANOVA (*P* < 0.05) with post hoc Tukey’s test *P* values shown. *P < 0.05 KF4 vs. IgG, ^#^*P* < 0.05 KF4 vs. unstretched. (**D**) Stretch-inducible lung elastase activity in mouse lung sections showing approximately 80% reduction in activity at all points. **P < 0.01, ***P < 0.001 by 2-tailed Welch’s *t* test. (**E**) Representative images showing elastase activity in mouse lung sections subjected to biaxial stretch with IgG and KF4 treatments.

We developed an anti-CELA1 monoclonal antibody by immunizing mice with 4 peptides corresponding to regions of human CELA1 with low mouse-human homology (Supplemental Table 2). Hybridomas were created, screened by ELISA, and KF4 was selected as our lead candidate based on inhibition of human lung elastase activity (Supplemental Figure 9). KF4 bound the N-terminal domain of CELA1 in a region including the histidine of the catalytic triad (Figure 8A) (26). Immunoprecipitation of human lung homogenate with the KF4 antibody identified both AAT-complexed and native CELA1 (Figure 8B). Since Cela1 was entirely responsible for stretch-induced lung elastase activity in mouse (6), we tested whether KF4 could reduce stretch-inducible lung elastase activity. In organ donor lung sections, KF4 reduced elastase activity to levels seen in unstretched lung (Figure 8C). In fresh mouse lung sections, KF4 reduced stretch-inducible lung elastase activity by 80% (Figure 8, D and E). While the levels of strain in these ex vivo assays exceed those seen physiologically, they provide proof of principle that antibody neutralization of CELA1 inhibits strain-induced elastase activity.

### KF4 penetrates lung tissue and prevents emphysema in 2 mouse models.

**Figure 9:**
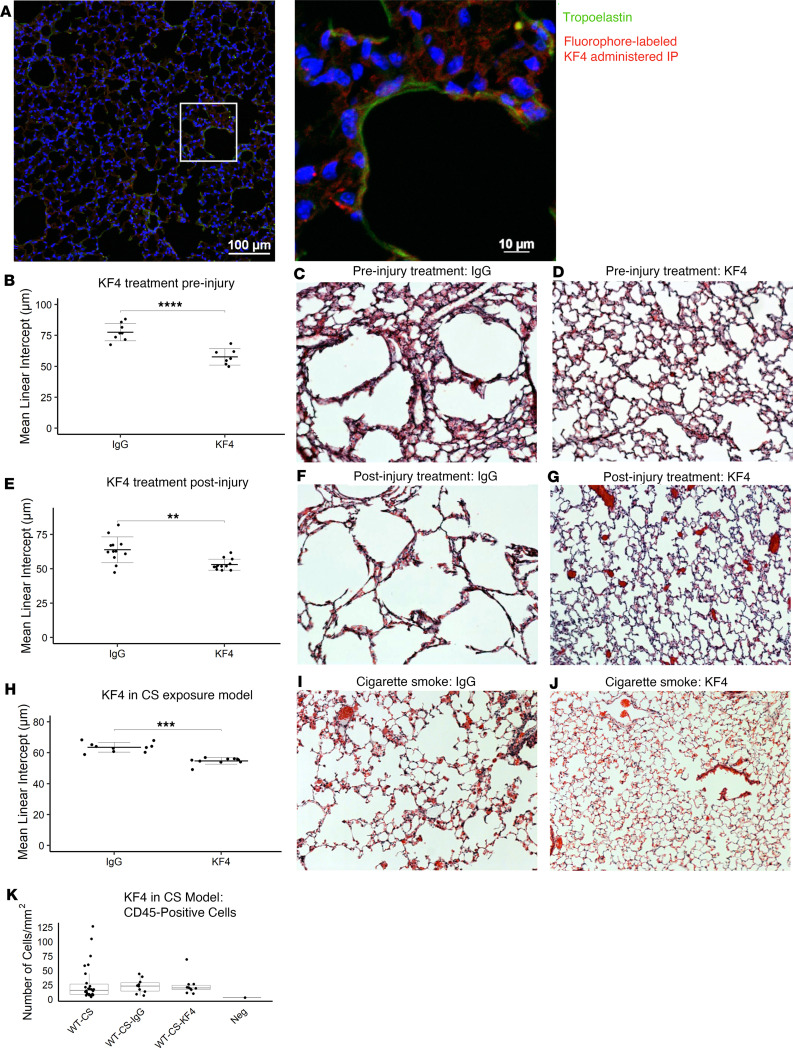
KF4 treatment in mouse models of alveolar simplification. (**A**) Left: Confocal image of a mouse that was administered fluorophore-labeled KF4 (red) at a dose of 5 mg/kg 24 hours before tissue harvest. The lung section was counterstained for tropoelastin (green). KF4 signal is present throughout the interstitium. Scale bar: 100 μm. Right: Magnified image showing a cell with increased binding of KF4, but also red signal throughout the lung matrix seemingly de-silhouetting cells suggested by central nuclei without red signal surrounding them. (**B**) Intraperitoneal administration of 5 mg/kg KF4 once weekly at the time of treatment with tracheal PPE resulted in a significant improvement in airspace simplification. Comparison by 2-tailed Welch’s *t* test. (**C**) Representative ×10 photomicrographs of IgG- and (**D**) KF4-treated mice are shown. (**E**) Initiating the same KF4 therapy 7 days after PPE administration still resulted in reduced airspace simplification. Comparison by 2-tailed Welch’s *t* test. (**F**) Representative ×10 photomicrographs of IgG- and (**G**) KF4-treated mice are shown. (**H**) KF4 administration to litter-matched WT mice exposed to cigarette smoke (CS) for 8 months reduced the amount of emphysema compared with IgG administration. *P* < 0.0001 by 1-way ANOVA and selected Tukey’s post hoc comparisons are shown. (**I**) Representative ×10 photomicrographs of IgG-treated and (**J**) KF4-treated mice are shown. (**K**) Quantitative image analysis of CS exposure alone, CS exposure with IgG treatment, and CS exposure with KF4 treatment showed no differences in the number of CD45-positive cells. Neg is analysis of secondary alone–treated lung section. **P < 0.01, ***P < 0.001, ****P < 0.00001.

To identify the dose of KF4 needed to reduce mouse lung elastolytic activity, we performed dosing studies in PPE-treated mice and identified an effective intraperitoneal dose of 5 mg/kg once weekly (Supplemental Figure 10). Using fluorophore-labeled KF4, we demonstrated penetration of KF4 into mouse lung tissue in a dose-dependent manner (Figure 9A and Supplemental Figure 10). Since *Cela1^–/–^* mice demonstrated late protection against emphysema in the PPE model, we compared emphysema in IgG- and KF4-treated mice beginning at the time of injury and 7 days after injury. The KF4 antibody prevented emphysema following tracheal PPE administration with both treatment strategies (Figure 9, B–G). After 8 months of 5 days per week CS exposure, mice treated with KF4 had less emphysema than mice treated with IgG (Figure 9, H–J). There was no significant difference in the number of lung immune cells between untreated, IgG-, and KF4-treated mice exposed to CS (Figure 9K). In summary, anti-CELA1 antibody KF4 penetrated lung tissue, inhibited stretch-inducible lung elastase activity, and preserved airspace architecture in the PPE and CS exposure models of alveolar simplification.

## Discussion

To the best of our knowledge, this is the first report of a role for *CELA1* in the strain-dependent emphysema progression in the peripheral lung. We also believe this to be the first report of a monoclonal antibody that protects against strain-induced alveolar simplification. *CELA1* may provide a mechanistic link between the protease-antiprotease and the structure-function models of emphysema. As *CELA1* is required for age-related alveolar simplification and elastin loss, it also links these 2 models to the accelerated aging model of COPD-associated emphysema.

We assert that the biology of CELA1 is distinct from that of other serine proteases and MMPs previously implicated in emphysema based on 3 findings. First, our correlative analysis of mRNA and activity levels in human lung showed no correlation of *CELA1* mRNA levels with those of other proteases except *MMP12*, and *CELA1* mRNA levels but not mRNAs of other proteases were correlated with lung elastase activity. Second, *CELA1* is expressed in AT2 cells (6, 27), while most emphysema-associated proteases are expressed in myeloid cells. Third, *Cela1^–/–^* mice were protected from emphysema differently than what was observed in other protease-focused emphysema studies. In those studies, inhibition or ablation of ELANE, CTSG, and MMP12 showed protection at 21 days or less after injury (28–31). In our study, we found that *Cela1* deletion or ablation was protective beyond 21 days. Consistent with our correlative analysis, others have found no correlation of MMP levels in bronchoalveolar lavage fluid and plasma with emphysema progression (32), although sputum MMP12 levels are elevated in COPD (33). Alveolar macrophage depletion protected mice against elastase-induced emphysema at 7, 14, and 21 days (34), and much work has shown the interaction of MMPs and immune cells in emphysema (35). Similar to emphysema models, in the mouse hyperoxia model of bronchopulmonary dysplasia (BPD), MMPs, neutrophil elastase, and immune cell activation have all been shown to be important (36–38). Most work in age-related airspace simplification has focused on senescence and altered inflammatory responses (39). Although in a sense this work on *CELA1* falls into the 50-year-old protease-antiprotease paradigm, the novelty of *CELA1* lies in the cell type in which it is expressed, in its being only recently described, and the biomechanical mechanism by which it works.

**Figure 10:**
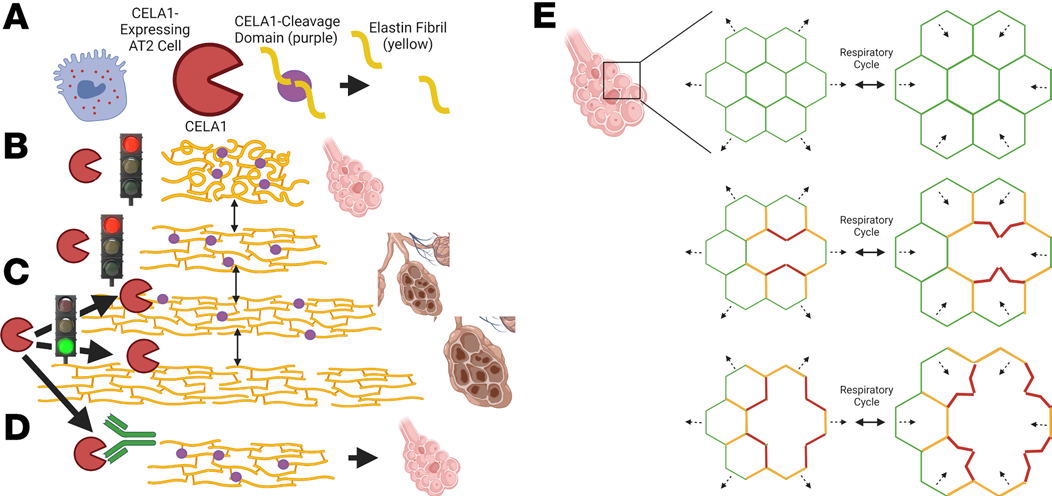
Mechanistic model of CELA1 in airspace simplification. (**A**) CELA1 (red) is expressed in AT2 cells. It cleaves the hydrophobic domains of tropoelastin (purple circles). (**B**) Under normal conditions, throughout the respiratory cycle, CELA1 proteolytic sites are not accessible. (**C**) Under conditions of increased strain, CELA1 proteolytic sites are accessible and CELA1 cleaves those elastin fibers. This leads to increased strain on adjacent fibers and more cleavage. (**D**) The KF4 antibody binds the CELA1 catalytic triad, prevents strain-induced remodeling, and preserves airspace architecture. (**E**) At the alveolar level, alveolar walls are modeled as a repeating geometric pattern. Under normal conditions, the strain of the respiratory cycle (dashed arrows) is evenly distributed (green = low strain). After destruction of an alveolar wall, there is a high level of strain in adjacent alveolar walls (red) and moderate strain 1 generation away (orange). High-strain walls are deformed, with respiration leading to CELA1-mediated elastolysis and destruction. This leads to a cycle of progressive airspace destruction and emphysema. This cycle can be arrested with KF4. Created with BioRender.com.

*CELA1* mechanistically links the protease-antiprotease and the biomechanical models of emphysema. CELA1 binds and cleaves non–cross-linked hydrophobic domains of elastin (6), and these domains are only exposed with strain. Our findings that (a) CELA1 binding to human lung elastin is enhanced by strain, (b) CELA1 inhibition reduces stretch-induced lung elastolytic activity, (c) *Cela1^–/–^* mice are protected from late but not initial airspace simplification, and (d) that postinjury treatment with KF4 is just as efficacious as preinjury treatment all support CELA1 as being a key mediator in localized strain-mediated airspace simplification. In this model, CELA1 is expressed by AT2 cells and binds and cleaves tropoelastin with strain (6). This increases strain in adjacent fibers, which in turn exposes additional proteolytic sites (Figure 10, A–C). KF4 binds the CELA1 catalytic domain and prevents elastolysis (Figure 10D). At the microstructural level, alveolar walls can be modeled as thin, geometrically shaped membranes under low strain suspended by thin elastic fibers — the fibers in the alveolar wall. These thin fibers are in turn supported by the more rigid fibers of the alveolar duct openings, which are interconnected and supported by progressively larger-caliber and less-compliant fibers of the small and large conducting airways and vessels and to the pleural surface. Strain is distributed over the entire network. Failure of one alveolar wall necessitates increased strain on adjacent walls, which both distorts local architecture and causes an imbalance of strain on additional alveolar walls. Each wall has a threshold beyond which it too will fail, imparting additional strain on its neighbors and predisposing them to failure (12, 14) (Figure 9E). The biomechanical model has been validated experimentally by showing that emphysema develops in regions of mouse lung not directly exposed to PPE (40). Furthermore, the role of *CELA1* in age-associated alveolar simplification is consistent with the accelerated aging model of COPD. If the same mechanisms in Figure 9 are operative in the aging lung, then *CELA1* provides a mechanistic link between 3 models: protease/antiprotease model, biomechanical, and accelerated aging.

Our study is not without limitations. Batch-to-batch variation in PPE injury precluded comparison between experiments. The biaxial strain model applies levels of strain that would not be experienced in the human lung. While we cannot definitively state that the same process would occur under physiological strain levels, neither can we perform this experiment over the months to years over which emphysema progresses. We cannot rule out the possibility that some absent role of *Cela1* in mouse lung development imparts resistance to postnatal lung injury; however, the protection conferred by anti-CELA1 antibody KF4 in multiple models makes this less likely. Lastly, we did not rigorously investigate whether *CELA1* could be upstream of other alveolar simplification programs, and it is possible that our in vivo effects are not direct ones.

### Conclusions.

The strain-dependent elastolytic activity of CELA1 is an important factor in airspace simplification at all stages of postnatal lung life, and neutralizing CELA1 with the KF4 antibody represents a potential therapy for BPD, COPD, and other disorders of progressive airspace simplification.

## Methods

### Animal use

Except for antibody production, all mice were on the C57BL/6 background, obtained from The Jackson Laboratory, and equal proportions of male and female mice used in all experiments. Mice were maintained in a barrier facility with access to food and water ad libitum and 12-hour light/dark cycles.

### Porcine pancreatic elastase model

Eight- to 10-week-old mice were anesthetized with 2% isoflurane and suspended on an intubating board by the front incisors. The trachea was cannulated with a 22-gauge angiocatheter and 2 units of porcine pancreatic elastase (Sigma-Aldrich, E1250) in 100 μL PBS was administered.

### CS model

Beginning at 10–12 weeks of age, matched littermate mice were exposed to 4 hours of CS 5 days per week for 8 or 10 months using a Teague TE-10z smoking machine using smoke generated from 3R4F Kentucky Reference Cigarettes (University of Kentucky) at a concentration of 150 mg/m^3^ total suspended particulates.

### Partial pneumonectomy model

Using previously described methods (41), the left lung of an anesthetized mouse was removed, mouse recovered, and tissues collected at 7 and 28 days.

### Aging model

Mice were sacrificed and collected at 72 to 75 weeks of age without any intervention.

### Mouse lung tissue

#### Tissue processing and morphometry.

After anesthetization and sacrifice by exsanguination, the mouse thorax was opened, left bronchus ligated, left lung snap frozen, trachea cannulated, right inflated with at 30 cmH_2_O pressure in 4% PBS, fixed overnight, lung lobes separated, and paraffinized. Lung lobes were randomly oriented and embedded, sectioned, and stained using hematoxylin and eosin. Using the methods of Dunnill (42), mean linear intercepts were determined in 5 fields per lobe. Left lungs were homogenized with extraction of RNA using RNeasy columns (Qiagen) or processed for protein analysis.

### Human lung tissue

COPD lung specimens were obtained from the National Heart, Lung, and Blood Institute Lung Tissue Consortium (now part of BioLINCC, Bethesda, Maryland), control and COPD specimens from the National Jewish Health Human Lung Tissue Consortium (William Jenssen, Denver, Colorado), and from The Ohio State University Human Lung Tissue Consortium (Meghan Baser, Columbus, Ohio).

### Enzymatic assays

Human lung protease, gelatinase, and elastase activity was quantified using Enzcheck fluorometric assays (Thermo Fisher Scientific) and elastase activity defined as rate of signal change. For comparative inhibition assays (i.e., antibody inhibition), the fractional change versus untreated was used for comparisons.

### Ex vivo lung stretch

Using a previously described lung stretching technique and device (6, 8, 43), the stretch-dependent binding of recombinant CELA1, albumin, and elastolytic activity of mouse and human lung sections was determined.

### Immunofluorescence, immunohistochemistry, and proximity ligation assay

For immunohistochemistry, the ABC Vectastain kit was used with a previously validated anti-CELA1 guinea pig polyclonal antibody (5). For immunofluorescence imaging, the KF4 anti-CELA1 antibody was used with Abcam anti-tropoelastin antibody (ab21600), both diluted 1:200. For proximity ligation assay, the Duolink Proximity Ligation Assay kit (Thermo Fisher Scientific) was used with these same 2 antibodies.

### Creation of lung single-cell suspension

After flushing collection, mouse lungs were digested by inflating with Dispase (BD Biosciences) and incubating for 45 minutes at 37°C. Afterward, lungs were transferred to a 100-cm^2^ petri dish with MEM and 10% FBS and minced removing the trachea and bronchi. The suspension was passed through a 70-μm strainer, pelleted, and resuspended in either MEM with 10% FBS or red cell lysis buffer (BD Biosciences) for colony-forming unit assay or flow cytometry, respectively.

### Flow cytometry

Cells (1 × 10^7^) were incubated (4°C, 30 minutes) with anti–mouse CD16/CD32 (BD Biosciences, 553142) to block Fc receptors. The cells were re-incubated (10 minutes, room temperature) with cell-surface markers (all diluted 1:100). For intracellular staining, cells were washed and fixed (room temperature, 30 minutes) with the True-Nuclear Transcription Factor Buffer set fixative (BioLegend) and permeabilized (4°C, overnight) using 1× Permeabilization Buffer according to the manufacturer’s instructions. Cells were stained with intracellular antibodies (diluted 1:100) and then washed (twice) and resuspended in flow cytometry buffer. Data were acquired with an Aurora (Cytek) and analyzed with FlowJo (Tree Star). Antibodies for flow cytometry are listed in Table 2.

### Lung cell colony-forming unit assay

Epithelial cells were purified from lung cell suspensions using CD326 (EpCAM) MicroBeads for mouse from Miltenyi Biotec. These cells were combined with postnatal day 10 mouse lung fibroblasts (isolated as previously described; ref. 44) in a 1:10 ratio in a 50:50 mixture of Matrigel (Corning) and MEM with 10% FBS. Six thousand epithelial and 60,000 lung fibroblasts were deposited in a 200 μL droplet in the bottom of a 24-well plate, allowed to solidify, and grown for 14 days. Droplets were imaged at ×4 magnification and the number of colonies counted.

### Imaging and quantitative image analysis

For immunofluorescent confocal imaging, a Nikon A2 inverted confocal microscope was used, and for bright-field imaging, a Nikon NiE microscope was used. For quantitative image analysis, ×10 magnified tile-scanned bright-field images were obtained and intensity masks applied to all images to identify CELA1-positive cells. The number of positive cells within an overlaid 1 mm × 1 mm grid and the distance between each cell and its nearest CELA1-positive cell was quantified. The average values for each lung section were used for comparison.

For quantification of CD45-positive cells in lung cell, mouse lung sections were stained for CD45 (Cell Signaling Technology, 70257s) and the VECTASTAIN Immunohistochemistry kit (Vector Laboratories). The ×4 tiled images were similarly analyzed, but total CD45-positive cells were normalized to lung tissue area by quantifying the area with saturation values above background.

### PCR

TaqMan PCR and SYBR Green PCR (both Thermo Fisher Scientific) were used for mouse and human lung PCR using primers in Supplemental Tables 3 and 4.

### Anti-CELA1 monoclonal antibody generation and screening

CD-1 female mice were immunized with CELA1 peptides (Supplemental Table 2) conjugated to CRM197. Each mouse received a primary, subcutaneous immunization of 20 μg of the conjugate with 50% Titermax Gold adjuvant (Sigma-Aldrich). The mice received subsequent immunizations of 20 μg (day 21) and 10 μg (day 35). Following the day 35 immunization, serum samples were obtained and tested for reactivity with CELA1. Based on the results of the serum titration, 1 mouse was selected for hybridoma production. The mouse received an intravenous immunization of 2 μg of conjugate and 3 days later the mouse was euthanized and the spleen excised for hybridoma formation with SP2/0 B lymphocytes from American Type Culture Collection (ATCC). Supernatants from the resulting hybridomas were tested for reactivity with CELA1 and were subsequently expanded and cloned. Antibodies from the cloned culture, derived from serum-free medium, were used in the studies reported here. Isotyping was performed using the Iso-Gold Rapid Mouse-Monoclonal Isotyping Kit (BioAssay Works, KSOT03-010).

### Immunoprecipitation and Western blotting

Ten milligrams of lung homogenate from 2 organ donors was incubated with 100 μg KF4 antibody and KF4 precipitated using Protein A/G beads, washed, and eluting using loading dye. Western blotting was performed using KF4 antibody, anti-AAT antibody (Abcam, ab133642), and anti-tropoelastin antibody (Abcam, ab21600). Revert total protein stain was used for normalization (LICOR, 926-11011). See complete unedited blots in the supplemental material.

### Study approval

Animal use was approved by the Cincinnati Children’s Hospital Medical Center Institutional Animal Use and Care Committee (2020-0054). For CS exposure experiments, mice were housed in the University of Cincinnati animal care facilities, and experimental procedures were performed in accordance with the Institutional Animal Care and Use Committee at the University of Cincinnati Medical Center (protocol AM06-20-10-28-01). Human tissue was utilized under a waiver from the Cincinnati Children’s Hospital Medical Center IRB (2016-9641).

### Statistics

Using R version 4.0.2 (45), the following packages were used for statistical comparisons and graphics generation: ggpubr (46), gridExtra (47), cowplot (48), ggplotify (49), corrplot (50), and rstatix (51). For parametric data, Welch’s *t* test and 1-way ANOVA with Holm-Šidák post hoc test were used, and for nonparametric data, Wilcoxon’s rank-sum and Kruskal-Wallis with Dunn’s post hoc test were used. All comparisons were 2-tailed. For correlative analyses, Pearson’s correlation with Bonferroni’s correction for multiple comparisons was used. Parametric data are displayed as line-and-whisker plots, with the center line representing the mean and whiskers standard deviation. Nonparametric data are displayed as box-and-whisker plots, with the center line representing the median value, boxes representing the 25th to 75th percentile range, and whiskers representing the 5th to 95th percentile range. For both plot plot types, dots represent individual data points. For all analyses, *P* values of less than 0.05 were considered significant.

### Data availability

The scripts and raw data for this these studies can be accessed at https://doi.org/10.6084/m9.figshare.24512995.v1 and are also available by contacting the corresponding author. Data values, means, medians, standard deviations, and percentile values used in plotting are summarized in the Supporting Data Values file.

## Author contributions

BMV made substantial contributions to the conception and design of the work. MO, NJS, AJD, RJ, EMG, QF, RS, AP, JMW, ET, MRB, JG, HD, MTB, SAA, and BMV made substantial contributions to the acquisition, analysis, or interpretation of data. MO, NJS, AJD, RJ, EMG, QF, RS, AP, JMW, ET, MRB, JG, HD, MTB, SAA, and BMV drafted the manuscript or revising it critically for important intellectual content. MO, NJS, AJD, RJ, EMG, QF, RS, AP, JMW, ET, MRB, JG, HD, MTB, SAA, and BMV approved the manuscript version to be published. MO, NJS, AJD, RJ, EMG, QF, RS, AP, JMW, ET, MRB, JG, HD, MTB, SAA, and BMV are accountable for all aspects of the work in ensuring that questions related to the accuracy or integrity of any part of the work are appropriately investigated and resolved.

## Supplementary Material

Supplemental data

Supporting data values

## Figures and Tables

**Table 1:**
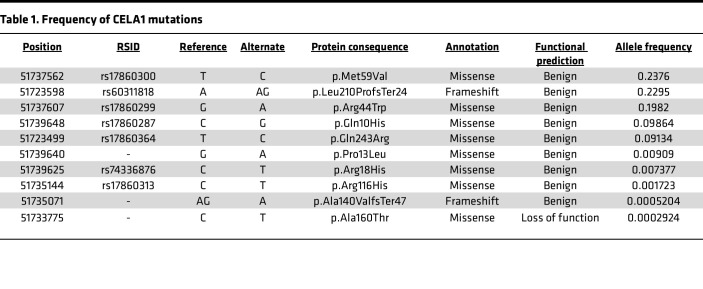
Frequency of CELA1 mutations

**Table 2:**
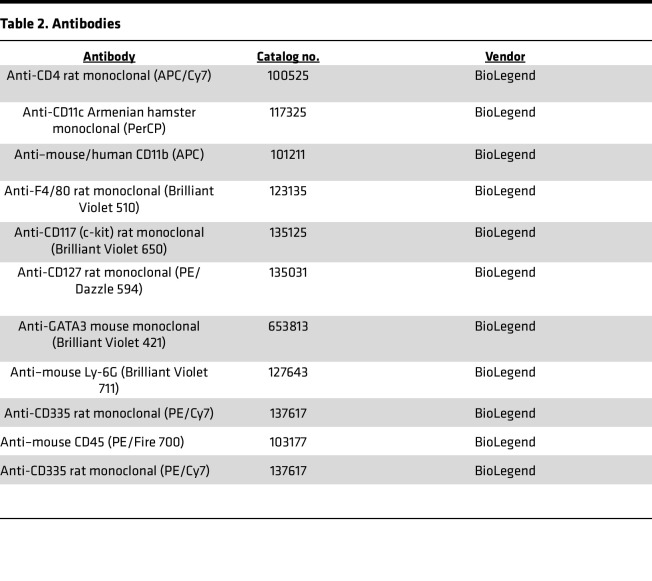
Antibodies

## Version 1 01/09/2024

Electronic publication

## References

[1] https://www.who.int/news-room/fact-sheets/detail/the-top-10-causes-of-death.

[2] Celli BR, Wedzicha JA (2019). Update on clinical aspects of chronic obstructive pulmonary disease. N Engl J Med.

[3] Ash SY (2021). Relationship between emphysema progression at CT and mortality in ever-smokers: results from the COPDGene and ECLIPSE cohorts. Radiology.

[4] Ozsvar J (2021). Tropoelastin and elastin assembly. Front Bioeng Biotechnol.

[5] Liu S (2014). Dynamic expression of chymotrypsin-like elastase 1 over the course of murine lung development. Am J Physiol Lung Cell Mol Physiol.

[6] Joshi R (2018). Role for Cela1 in postnatal lung remodeling and alpha-1 antitrypsin-deficient emphysema. Am J Respir Cell Mol Biol.

[7] Bird AD (2014). Mesenchymal glucocorticoid receptor regulates development of multiple cell layers of the mouse lung. Am J Respir Cell Mol Biol.

[8] Joshi R (2016). Stretch regulates expression and binding of chymotrypsin-like elastase 1 in the postnatal lung. FASEB J.

[9] Laurell CB, Eriksson S (1964). [Hypo-alpha-1-antitrypsinemia]. Verh Dtsch Ges Inn Med.

[10] Mercado N (2015). Accelerated ageing of the lung in COPD: new concepts. Thorax.

[11] Parameswaran H (2011). Linking microscopic spatial patterns of tissue destruction in emphysema to macroscopic decline in stiffness using a 3D computational model. PLoS Comput Biol.

[12] Hamakawa H (2011). Structure-function relations in an elastase-induced mouse model of emphysema. Am J Respir Cell Mol Biol.

[13] Ito S (2005). Mechanics, nonlinearity, and failure strength of lung tissue in a mouse model of emphysema: possible role of collagen remodeling. J Appl Physiol (1985).

[14] Suki B (2013). Emphysema and mechanical stress-induced lung remodeling. Physiology (Bethesda).

[15] Baker JR (2019). MicroRNA-570 is a novel regulator of cellular senescence and inflammaging. FASEB J.

[16] Wells JM (2019). The matrikine acetyl-proline-glycine-proline and clinical features of COPD: findings from SPIROMICS. Respir Res.

[17] Wells JM (2018). Elevated circulating MMP-9 is linked to increased COPD exacerbation risk in SPIROMICS and COPDGene. JCI Insight.

[18] Devine AJ (2023). Chymotrypsin-like elastase-1 mediates progressive emphysema in alpha-1 antitrypsin deficiency. Chronic Obstr Pulm Dis.

[19] Jesudason R (2010). Mechanical forces regulate elastase activity and binding site availability in lung elastin. Biophys J.

[20] Sakornsakolpat P (2019). Genome-wide association analysis of single breath diffusing capacity of carbon monoxide (DLCO). Am J Respir Cell Mol Biol.

[21] Genomes Project Consortium (2015). A global reference for human genetic variation. Nature.

[22] https://www.ncbi.nlm.nih.gov/gap/.

[23] Landrum MJ (2014). ClinVar: public archive of relationships among sequence variation and human phenotype. Nucleic Acids Res.

[24] Adzhubei I (2013). Predicting functional effect of human missense mutations using PolyPhen-2. Curr Protoc Hum Genet.

[25] Sim N-L (2012). SIFT web server: predicting effects of amino acid substitutions on proteins. Nucleic Acids Res.

[26] Bienert S (2017). The SWISS-MODEL repository-new features and functionality. Nucleic Acids Res.

[27] Du Y (2015). ‘LungGENS’: a web-based tool for mapping single-cell gene expression in the developing lung. Thorax.

[28] Haq I (2011). Matrix metalloproteinase-12 (MMP-12) SNP affects MMP activity, lung macrophage infiltration and protects against emphysema in COPD. Thorax.

[29] Guyot N (2014). Unopposed cathepsin G, neutrophil elastase, and proteinase 3 cause severe lung damage and emphysema. Am J Pathol.

[30] Shipley JM (1996). Metalloelastase is required for macrophage-mediated proteolysis and matrix invasion in mice. Proc Natl Acad Sci U S A.

[31] Suki B (2017). Elastase-induced lung emphysema models in mice. Methods Mol Biol.

[32] D’Armiento JM (2013). Increased matrix metalloproteinase (MMPs) levels do not predict disease severity or progression in emphysema. PLoS One.

[33] Demedts IK (2006). Elevated MMP-12 protein levels in induced sputum from patients with COPD. Thorax.

[34] Ueno M (2015). Alendronate inhalation ameliorates elastase-induced pulmonary emphysema in mice by induction of apoptosis of alveolar macrophages. Nat Commun.

[35] Churg A (2012). Series “matrix metalloproteinases in lung health and disease”: matrix metalloproteinases in COPD. Eur Respir J.

[36] Ambalavanan N (2008). Role of matrix metalloproteinase-2 in newborn mouse lungs under hypoxic conditions. Pediatr Res.

[37] Chetty A (2008). Role of matrix metalloprotease-9 in hyperoxic injury in developing lung. Am J Physiol Lung Cell Mol Physiol.

[38] Hilgendorff A (2012). Neonatal mice genetically modified to express the elastase inhibitor elafin are protected against the adverse effects of mechanical ventilation on lung growth. Am J Physiol Lung Cell Mol Physiol.

[39] Brandsma C-A (2017). Lung ageing and COPD: is there a role for ageing in abnormal tissue repair?. Eur Respir Rev.

[40] Sato S (2015). Scale dependence of structure-function relationship in the emphysematous mouse lung. Front Physiol.

[41] Liu S (2014). Mouse pneumonectomy model of compensatory lung growth. J Vis Exp.

[42] Dunnill MS (1962). Quantitative methods in the study of pulmonary pathology. Thorax.

[43] Young SM (2015). Localization and stretch-dependence of lung elastase activity in development and compensatory growth. J Appl Physiol (1985).

[44] Joshi R (2022). Mouse lung organoid responses to reduced, increased, and cyclic stretch. Am J Physiol Lung Cell Mol Physiol.

[45] https://www.R-project.org/.

[46] http://www.sthda.com/english/articles/24-ggpubr-publication-ready-plots/.

[47] https://cran.r-project.org/web/packages/gridExtra/index.html.

[48] https://wilkelab.org/cowplot/.

[49] https://cran.r-project.org/web/packages/ggplotify/vignettes/ggplotify.html.

[50] https://cran.r-project.org/web/packages/corrplot/corrplot.pdf.

[51] https://CRAN.R-project.org/package=rstatix.

