# Supplementary material for: Anti-CELA1 antibody KF4 prevents emphysema by inhibiting stretch-mediated remodeling
Source: JCI Insight. 2024 Jan 9;9(1):e169189. doi: 10.1172/jci.insight.169189 (PMC10906462; doi:10.1172/jci.insight.169189)
Supplement: Supplemental data [file jciinsight-9-169189-s055.pdf]

Supplemental Table 1: Human Subject Demographics

Supplemental Table 1: Subject Demographic Characteristics							
Specimen_ID	Source	Group	Age (years)	Sex	Current Smoker	Pack Years	Other
15215	BioLINCC/LTRC	Non-Smoker	80	Female	Unknown	Unknown	Old Age specimen
15473	BioLINCC/LTRC	COPD	66	Female	Unknown	Unknown	GOLD 3, R lower
40985	BioLINCC/LTRC	Non-Smoker	47	Female	Unknown	Unknown	R lower
94308	BioLINCC/LTRC	Non-Smoker	67	Male	Unknown	Unknown	R lower
107367	BioLINCC/LTRC	Former Smoker	80	Male	Unknown	Unknown	R lower
180982	BioLINCC/LTRC	Non-Smoker	51	Male	Unknown	Unknown	R lower
181462	BioLINCC/LTRC	Smoker	54	Female	Unknown	Unknown	R lower
185233	BioLINCC/LTRC	Smoker	58	Female	Unknown	Unknown	R middle
191599	BioLINCC/LTRC	Non-Smoker	72	Male	Unknown	Unknown	AAT project, Geno MS
195939	BioLINCC/LTRC	Former Smoker	57	Male	Unknown	Unknown	R lower
203575	BioLINCC/LTRC	Non-Smoker	62	Female	Unknown	Unknown	L lingula
204969	BioLINCC/LTRC	COPD	68		Unknown	Unknown	GOLD 4, R lung
216921	BioLINCC/LTRC	Former Smoker	61	Male	Unknown	Unknown	L upper
221228	BioLINCC/LTRC	Non-Smoker	69	Male	Unknown	Unknown	L upper
244293	BioLINCC/LTRC	Non-Smoker	63	Female	Unknown	Unknown	R upper
249864	BioLINCC/LTRC	Non-Smoker	61	Female	Unknown	Unknown	L lower
254654	BioLINCC/LTRC	Smoker	42	Male	Unknown	Unknown	R lower
274080	BioLINCC/LTRC	Non-Smoker	60	Male	Unknown	Unknown	R lower
274496	BioLINCC/LTRC	COPD	76		Unknown	Unknown	GOLD 2, R lung
282998	BioLINCC/LTRC	Non-Smoker	74	Female	Unknown	Unknown	R upper
289294	BioLINCC/LTRC	COPD	56		Unknown	Unknown	GOLD 2 R lower
300009	BioLINCC/LTRC	Former Smoker	73	Male	Unknown	Unknown	R lower
300020	BioLINCC/LTRC	Smoker	55	Male	Unknown	Unknown	L lung

400012	BioLINCC/LTRC	Non-Smoker	58	Male	Unknown	Unknown	R lower
400033	BioLINCC/LTRC	Non-Smoker	44	Male	Unknown	Unknown	
400054	BioLINCC/LTRC	Non-Smoker	44	Female	Unknown	Unknown	R lower
400056	BioLINCC/LTRC	Non-Smoker	74	Female	Unknown	Unknown	R middle
500015	BioLINCC/LTRC	Smoker	42	Female	Unknown	Unknown	L upper
118781	BioLINCC/LTRC	COPD	59		Unknown	Unknown	GOLD 3, R lower
294945	BioLINCC/LTRC	COPD	52		Unknown	Unknown	GOLD 4 R lung
NJ 25	National Jewish	COPD	50	Male	No	Unknown	PF ratio 470
NJ 5	National Jewish	COPD	56	Male	Yes	Unknown	PF ratio 321
NJ 10	National Jewish	Non-Smoker	18	Female	No	0	PF ratio 427
NJ 11	National Jewish	Non-Smoker	59	Female	No	0	PF ratio 263
NJ 12	National Jewish	Non-Smoker	87	Male	No	0	
NJ 13	National Jewish	Non-Smoker	51	Female	No	0	
NJ 14	National Jewish	Non-Smoker	51	Female	No	0	
NJ 15	National Jewish	Non-Smoker	61	Male	No	0	
NJ 16	National Jewish	Non-Smoker	66	Male	No	0	PF ratio 357
NJ 17	National Jewish	Non-Smoker	26	Male	No	0	PF ratio 343
NJ 2	National Jewish	Non-Smoker	26	Male	Yes	1	PF ratio 220
NJ 26	National Jewish	Non-Smoker	46	Male	No	0	PF ratio 308
NJ 3	National Jewish	Non-Smoker	35	Male	No	0	PF ratio 280
NJ 6	National Jewish	Non-Smoker	35	Female	No	0	PF ratio 370
NJ 8	National Jewish	Non-Smoker	39	Male	No	0	PF ratio 270
NJ 9	National Jewish	Non-Smoker	49	Male	No	0	
NJ 18	National Jewish	Smoker	28	Male	Yes	Unknown	PF ratio 220
NJ 19	National Jewish	Smoker	32	Male	Yes	Unknown	PF ratio 262
NJ 20	National Jewish	Smoker	50	Female	Yes	Unknown	PF ratio 481

NJ 23	National Jewish	Smoker	61	Female	Yes	Unknown	PF ratio 379
NJ 24	National Jewish	Smoker	66	Female	Yes	Unknown	PF ratio 313
NJ 4	National Jewish	Smoker	43	Female	Yes	Unknown	PF ratio 241
NJ 7	National Jewish	Smoker	44	Male	Yes	Unknown	PF ratio 293
NJ21	National Jewish	Smoker	63	Male	Yes	Unknown	PF ratio 379
NJ22	National Jewish	Smoker	62	Female	Yes	Unknown	PF ratio 493
OSU-200350	Ohio State	Non-Smoker	63	Male	No	0	Myocardial Infarction, Aspiration pneumonia
OSU-200360	Ohio State	Non-Smoker	52	Male	No	0	Abdominal Aortic Aneurysm
Abbreviations: LTRC=Lung Tissue Research Consortium NJ=National Jewish OSU=Ohio State University PF=PaO2/FiO2 AAT = Alpha-1 Antitrypsin, Geno = Genotype MS=M-allele and S-Allele for AAT							

Supplemental Table 2: Human CELA1 Peptides

Supplemental Table 2: hCELA1 Peptides	
hCELA1 (30-54)	CGTEAGRNSWPSQISLQYRSGGSRYH
hCELA1 (62-86)	CRQNWVMTAAHCVDYQKTFRVVAGDH
hCELA1 (104-134)	CVVHPYWNSDNVAAGYDIALLRLAQSVTLNSY
hCELA1 (159-183)	CGKTKTNGQLAQTLQQAYLPSVDYAI
hCELA1 (220-244)	CLVNGKYSVHGVTSFVSSRGCNVSR

Supplemental Table 3: Taqman Primers

Supplemenal Table 3: Taqman Primers	
Target	Primer Catalog Number
Human Matrix Metaloproteinase-2	4331182_Hs01548727
Human Matrix Metaloproteinase-8	4453320-Hs01029057
Human Matrix Metaloproteinase-9	4453320-Hs00957562_m1
Human Matrix Metaloproteinase-12	4448892-Hs00159178
Human Matrix Metaloproteinase-14	4448892-Hs01037003
Human Proteinase-3	4448892-Hs01553330_m1
Human Neutrophil Elastase	4331182-Hs00236952_m1
Human Chymotrypsin-like Elastase 1	4331182Hs00608115_m1
Human Cathepsin G	4448892-Hs00175195_m1
Eukaryotic 18S RNA	4333760T

Supplemental Table 4: SybrGreen Primers

Supplemenal Table 4: SybrGreen Primers	
Name	Sequence
MsCela1 FL1-Fwd	TTGTCGGAGAGCACAACCTG
MsCela1 FL1-Rev	CCAAGACACCAGCAGCATTC
MsGapdh(66-323) mRNA-Fwd	AGAGTGTTTCCTCGTCCCGT
MsGapdh(66-323) mRNA-Rev	TGATGTTAGTGGGGTCTCGC

Supplemental Figure Legends

Supplemental Figure 1

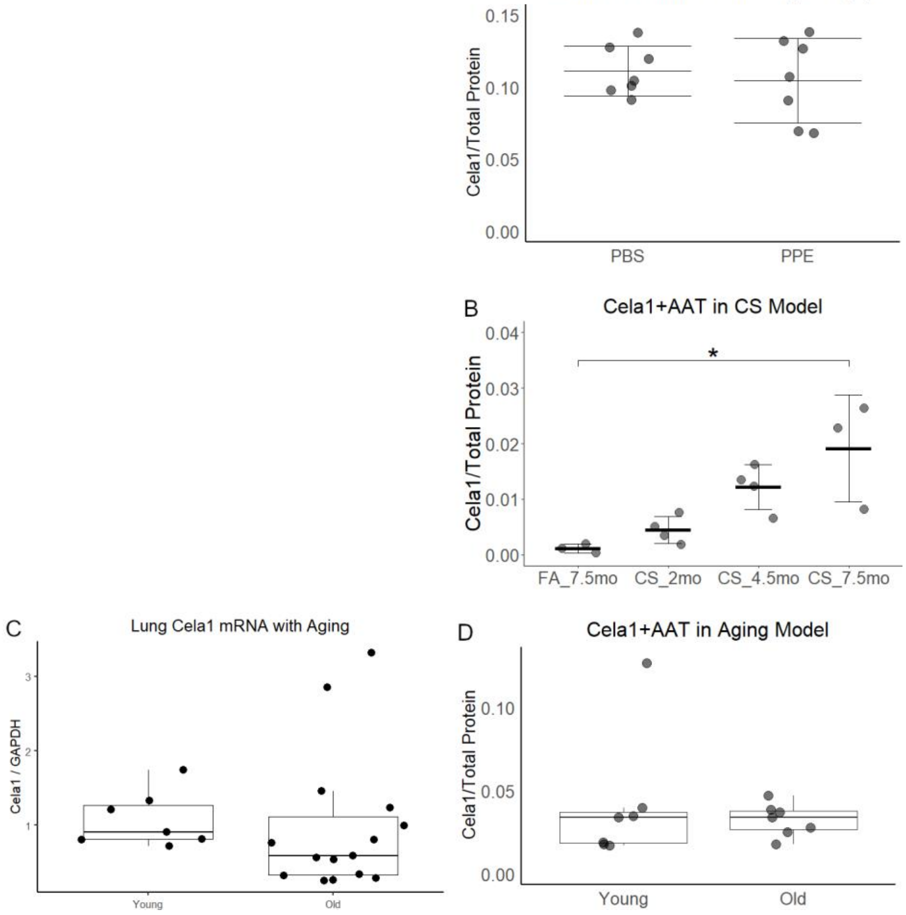

Supplemental Figure 1: Chymotrypsin-like Elastase 1 Expression in Three Mouse Models of Emphysema-Supplemental Data. (A) There was no difference in the amount of high molecular weight Cela1 (previously shown to be Cela1 neutralized by alpha-1 antitrypsin, Cela1+AAT) at 42 days after porcine pancreatic elastase (PPE) compared to phosphate buffered saline (PBS). (B) The amount of high molecular weight Cela1 increased with duration of cigarette smoke (CS) exposure compared to filtered air (FA). ANOVA $p<0.05$, Holm Sidak *post hoc* test $*p<0.05$. (C) Normalized *Cela1* mRNA levels were no different in the aged (72-75 week old) mice compared to young (8-10 week). (D) There was also no difference in the amount of high molecular weight Cela1 protein.

A

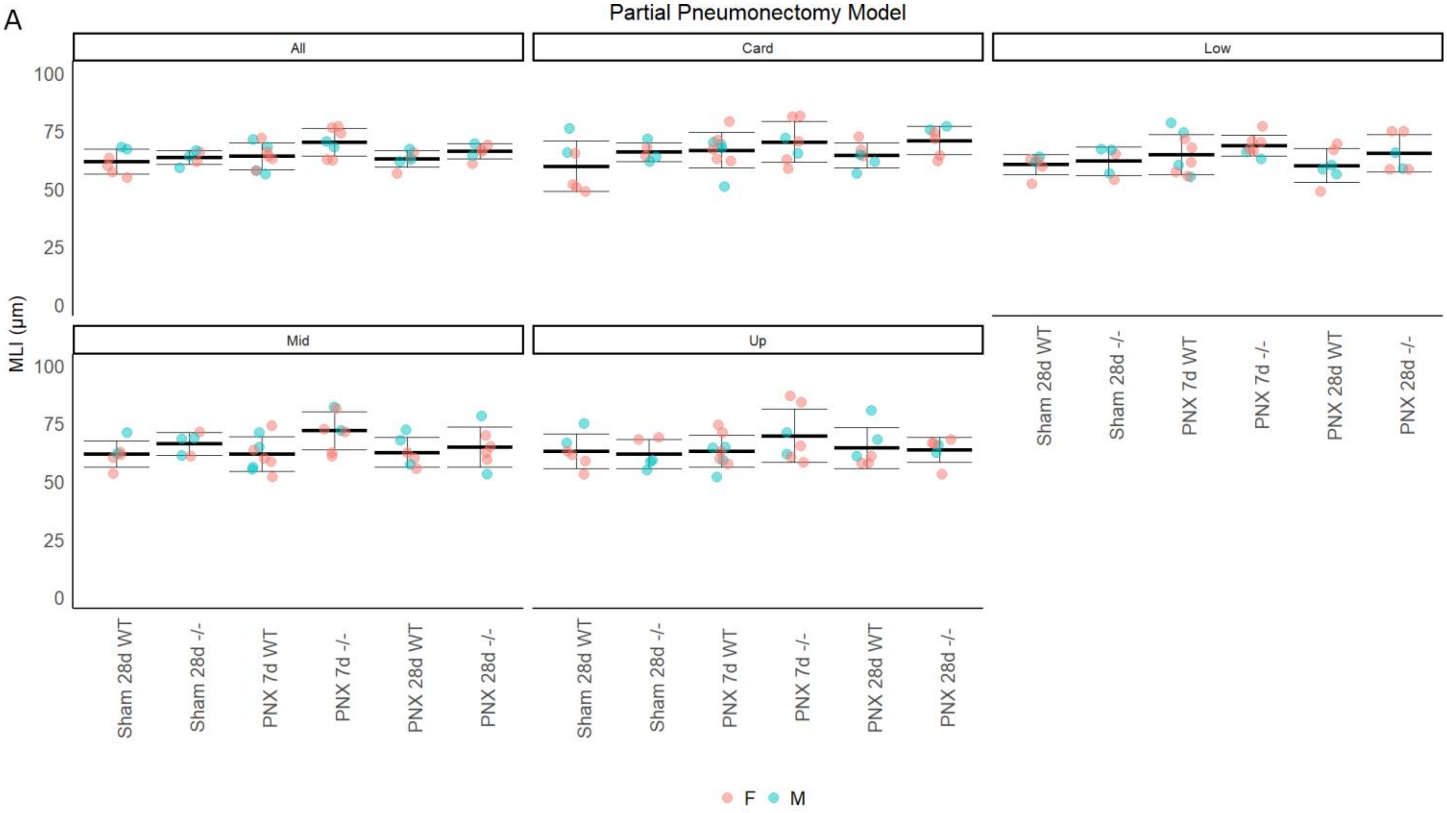

B

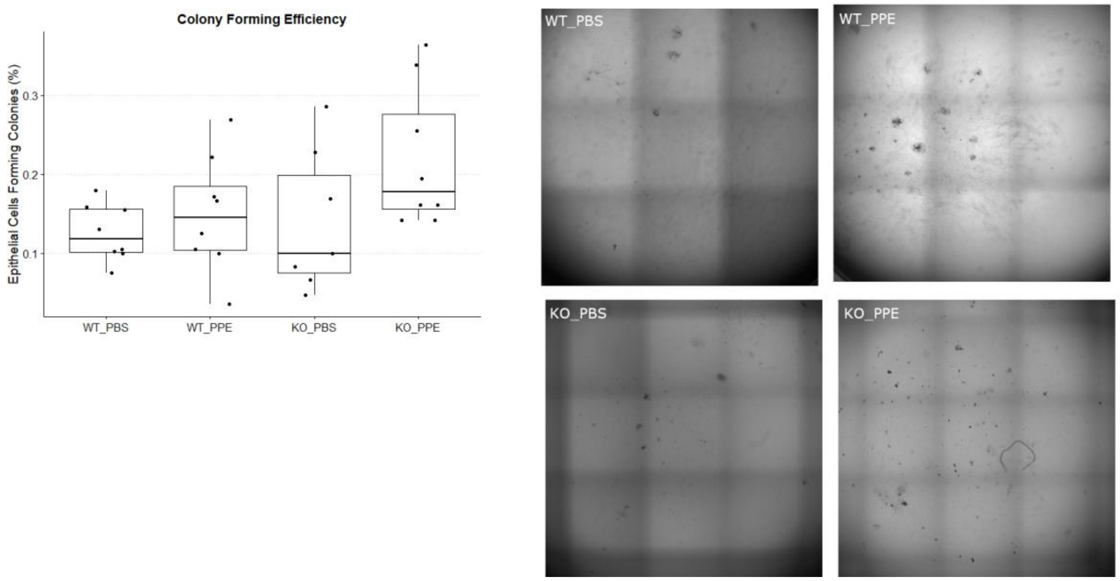

Supplemental Figure 2: No Impact of CELA1 in Compensatory Lung Re-growth after Partial Pneumonectomy. (A) Wildtype (WT) and *Cela1*$^{-/-}$ (-/-) mice were subjected to left lung partial pneumonectomy, and the airspace size of the remaining lung lobes quantified at 7 and 28 days. Mean linear intercepts (MLI) were compared to Sham at 28 days. A small increase in MLI in *Cela1*$^{-/-}$ mice at 7 days was not significant and normalized by 28 days. (B) Colony forming unit assays on epithelial cells from WT and *Cela1*$^{-/-}$ (KO) mice did not demonstrate any significant differences in the number of colonies, but there was a trend towards greater numbers in KO mice treated with PPE.

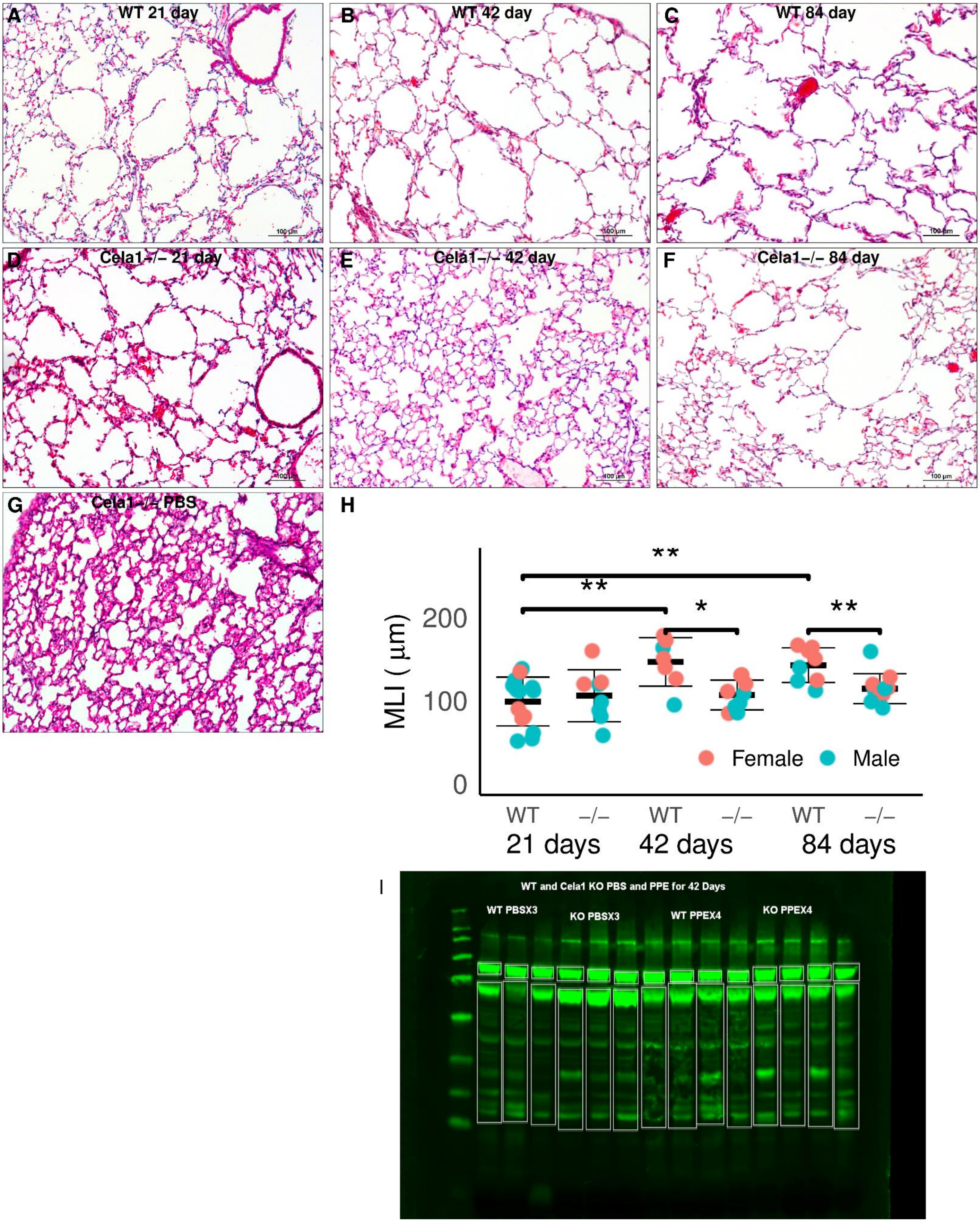

Supplemental Figure 3: Protection of *Cela1*$^{-/-}$ Mice in PPE Model. (A) Mice were treated with 2 units of PPE instilled into the posterior nasopharynx. Treated wildtype (WT) mice had evidence of airway simplification at 21 days. 10X photomicrograph, scale bar = 100 µm. (B) This airspace simplification was worse at 42 days and (C) at 84 days. (D) *Cela1*$^{-}$

$^{/-}$ mice had a similar degree of airspace injury at 21 days. (E) However, at 42 and (F) 84 days, these mice did not experience progression of airspace simplification. (G) PBS-treated *Cela1*$^{-/-}$ mice had normal appearing airspace architecture. (H) Line and whisker plot showing the above differences. There was no difference in emphysema by sex. ANOVA $p < 0.01$. Tukey post hoc comparisons are shown. (I) Tropoelastin Western blot of mouse lung homogenates with densitometry quantification blocks showing intact (higher molecular weight box) and degraded (elongated, lower box) tropoelastin. *$p<0.05$, **$p<0.01$.

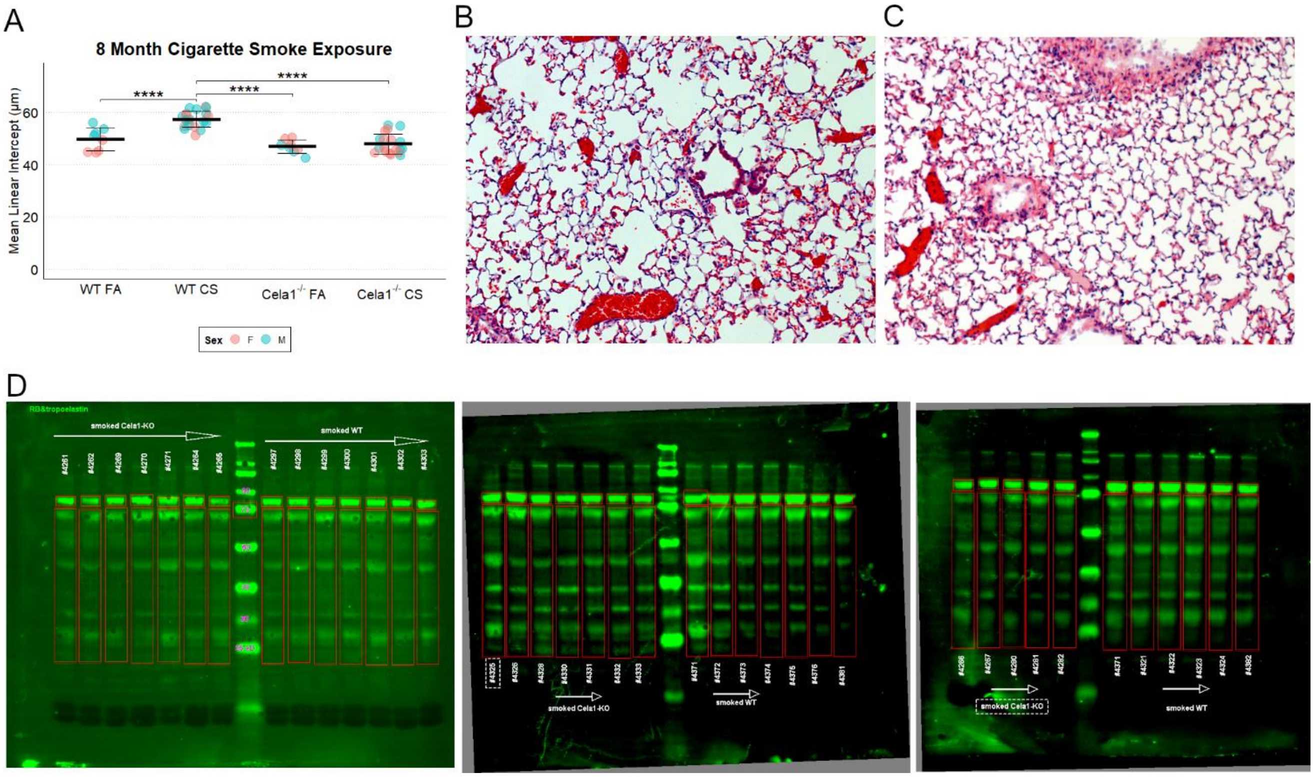

Supplemental Figure 4: Protection of *Cela1*$^{-/-}$ Mice in Cigarette Smoke Model of Emphysema. (A) Compared to wildtype (WT) mice, *Cela1*$^{-/-}$ mice had no difference in airspace size at baseline but were protected against airspace simplification in response to cigarette smoke (CS) exposure. There was no impact of sex on airspace size. ANOVA p<0.00001. Tukey *post hoc* comparisons are shown. (B) Representative 10X photomicrograph of CS-exposed WT lung and (C) CS-exposed *Cela1*$^{-/-}$ lung. (D) Three Western blot images of lung homogenates for quantification of intact (higher molecular weight box) and degraded (elongated, lower box) tropoelastin.

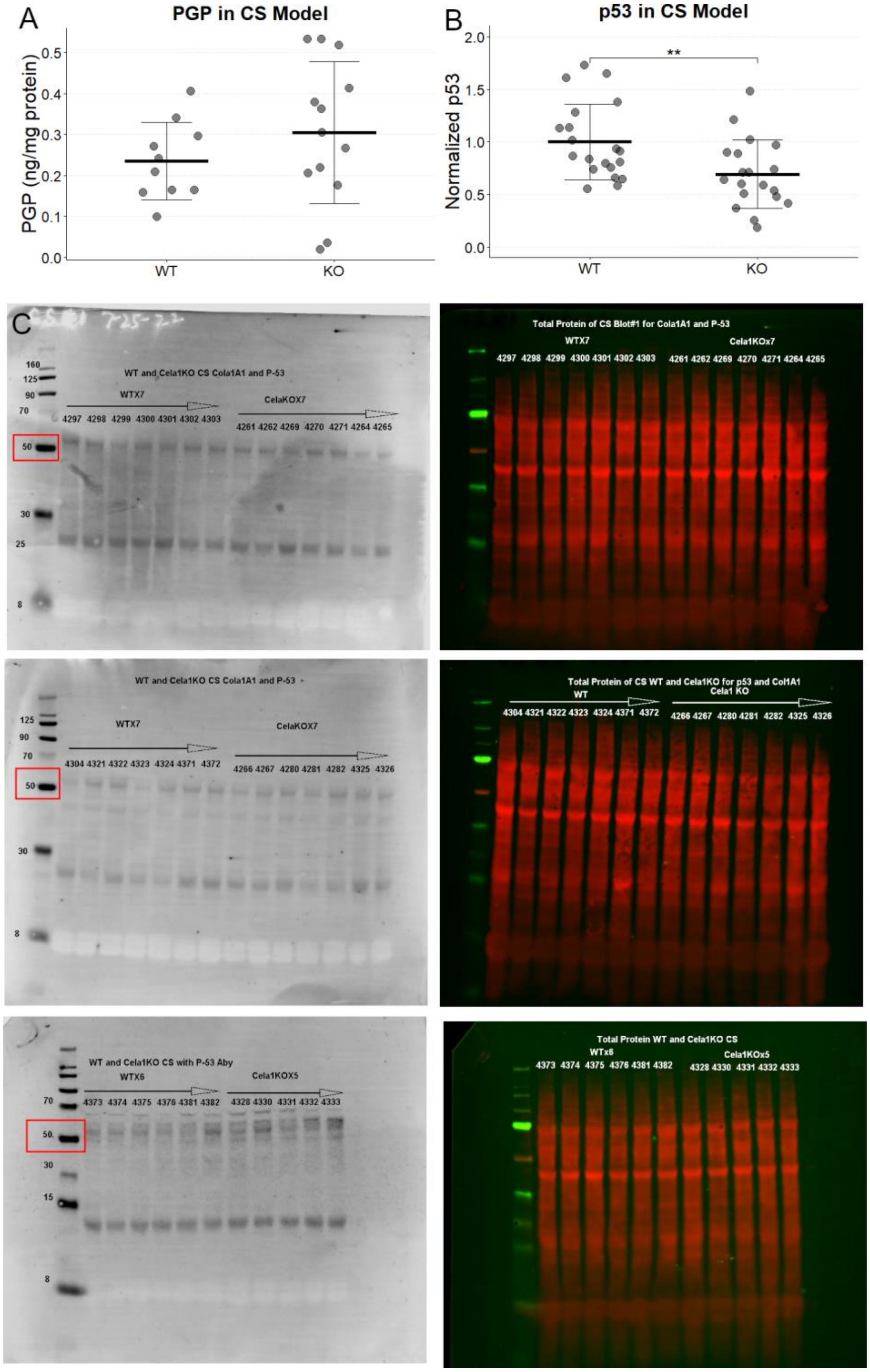

Supplemental Figure 5: Collagen and p53 in the CS Model. (A) Using mass spectrometry to quantify the collagen matrikine proline-glycine-proline (PGP), there were no significant differences between wild type and *Cela1*$^{-/-}$ mice after cigarette smoke exposure. (B) The senescence marker p53 was lower in the lungs of *Cela1*$^{-/-}$ mice exposed to cigarette smoke. Comparison by Welch's t-test. **p<0.01. (C) Image blots of p53 on left and total protein on right to which signal was normalized. The red box highlights the 50 kDa molecular weight maker.

Supplemental Figure 6: Immune Cell Flow Cytometry Data in *Cela1*$^{-/-}$ Mice Treated with Tracheal Porcine Pancreatic Elastase. (A) A total of 250,000 cells were analyzed. Surface and cytoplasmic markers for each immune cell population showed that while most leukocytes were T-cells, the difference in T-cell numbers was not significant. The absolue numbers of neutrophils and eosinophils was significantly increased. (B) In evaluating the fraction of total leukocytes that each population accounted for, we also found that neutrophils and eosinophils were increased. *$p<0.05$, **$p<0.01$ by Dunn's *post hoc* test after Kruskal-Wallis test $p<0.05$.

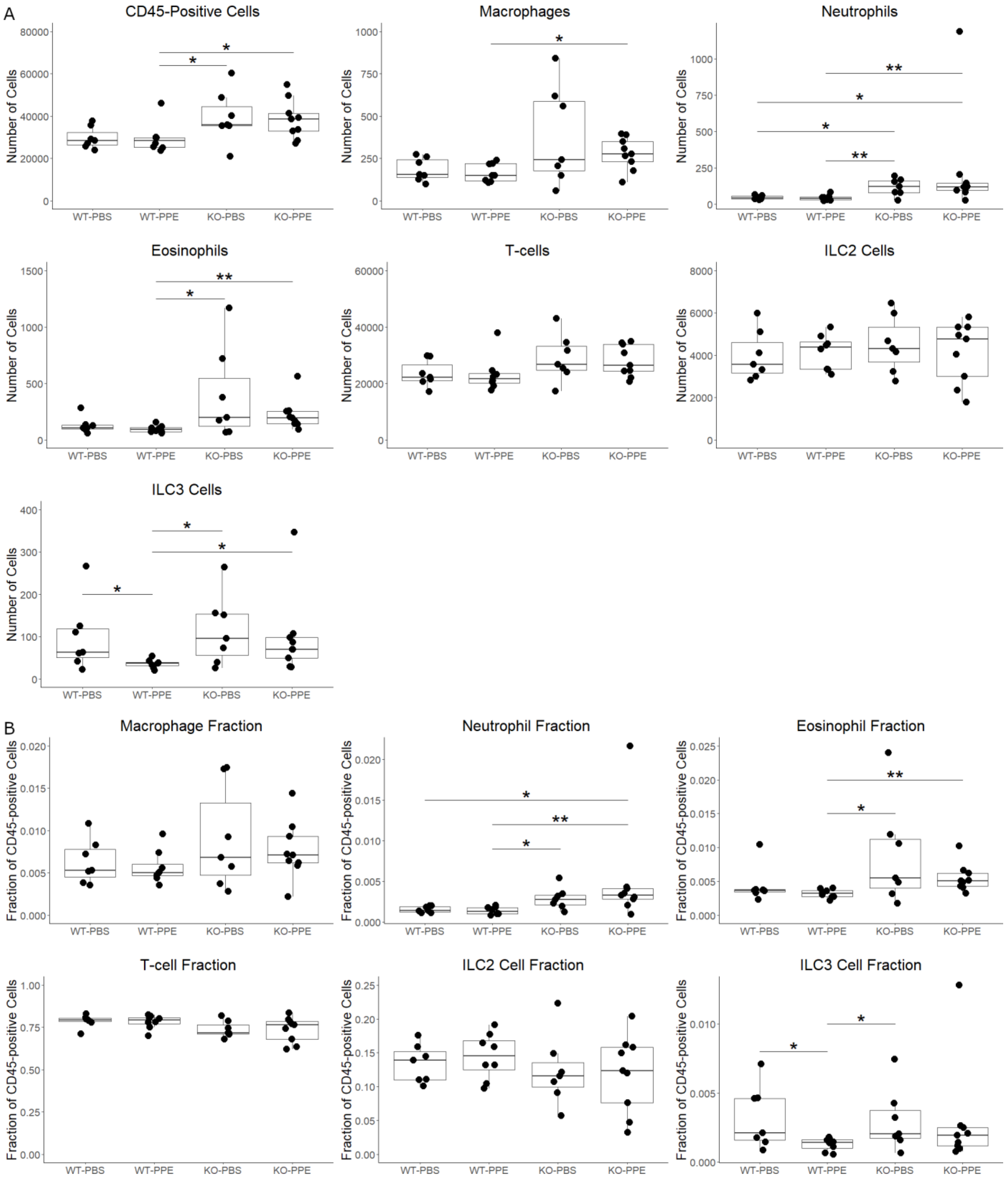

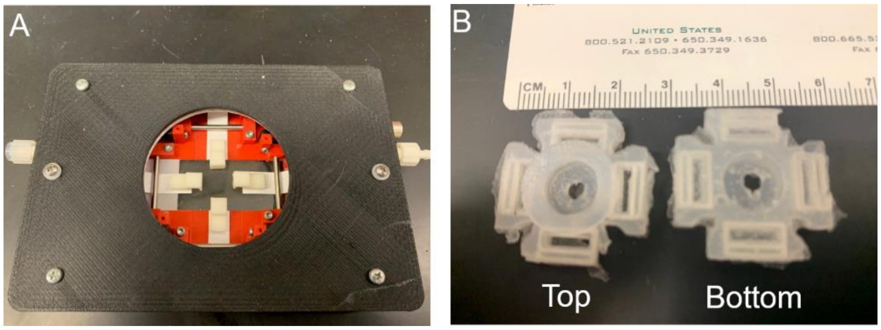

Supplemental Figure 7: Biaxial Stretch of Human Lung Tissue. (A) Image of a 3D-printed, confocal microscope stretching device. The device fits into a K-mount of a Nikon A1 confocal microscope and contains 4 small motors which incrementally turn a Tyvek strip to which is attached printed clips. These clips are secured to a silicone mount. (B) Silicone mount which is created using 3D-printed molds with embedded eyelets to which the clips are secured. 100 µm sections of human lung are secured to the underside of the mount at four points using surgical glue.

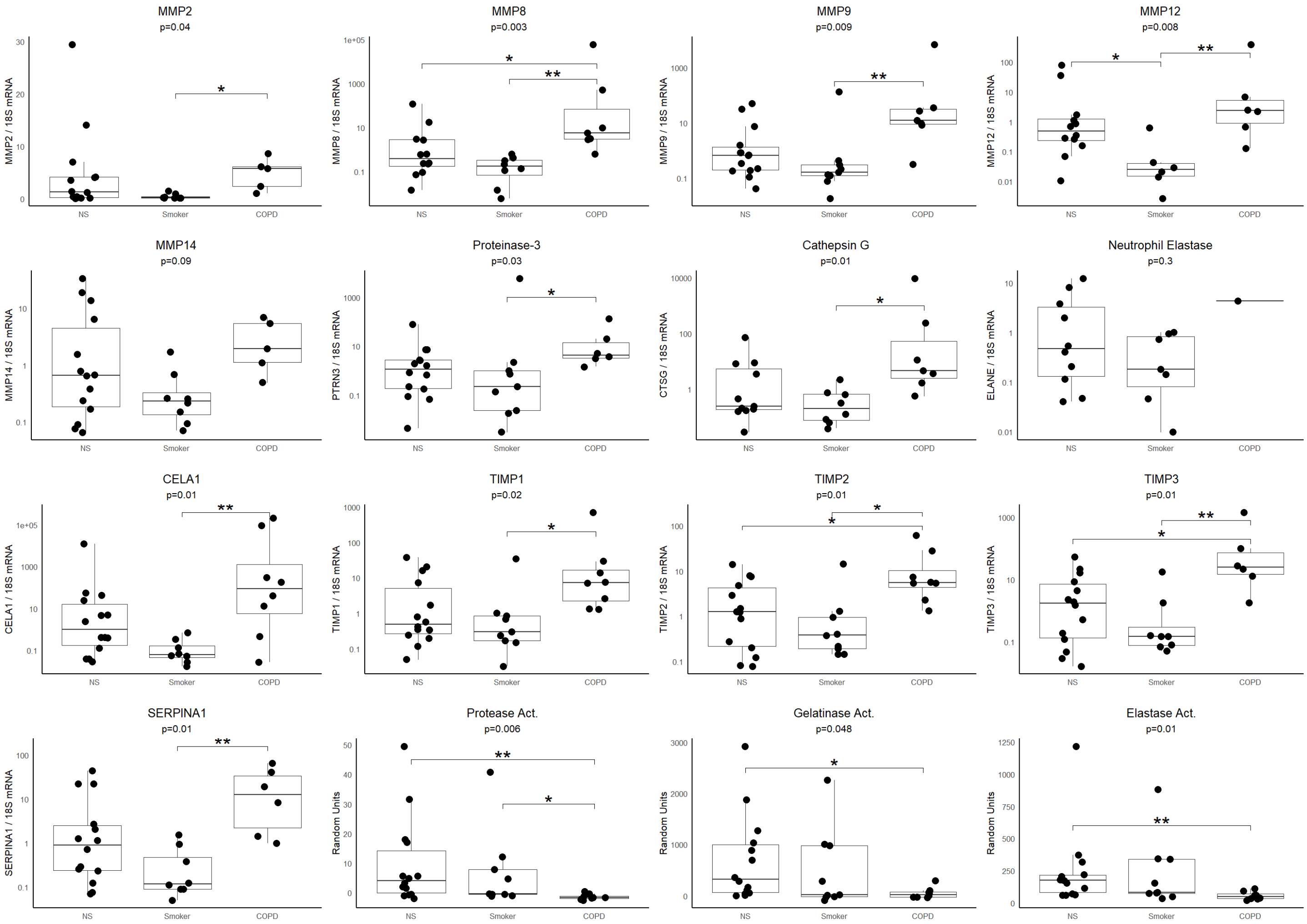

Supplemental Figure 8: mRNA Levels of COPD-associated Proteases and Anti-Proteases. The mRNA levels of matrix metalloproteinase 2 (MMP2), MMP8, MMP9, MMP12, MMP14, Proteinase-3, Cathepsin G, Neutrophil Elastase, CELA1, Tissue Inhibitor of Metalloproteinase-1 (TIMP1), TIMP2, TIMP3, and α1-antitrypsin (SERPINA1) were generally higher in COPD than smoker controls and non-smoker (NS) controls. Tissue protease, gelatinase, and elastase activities were all lower in COPD however. Kruskal-Wallis p values are shown.

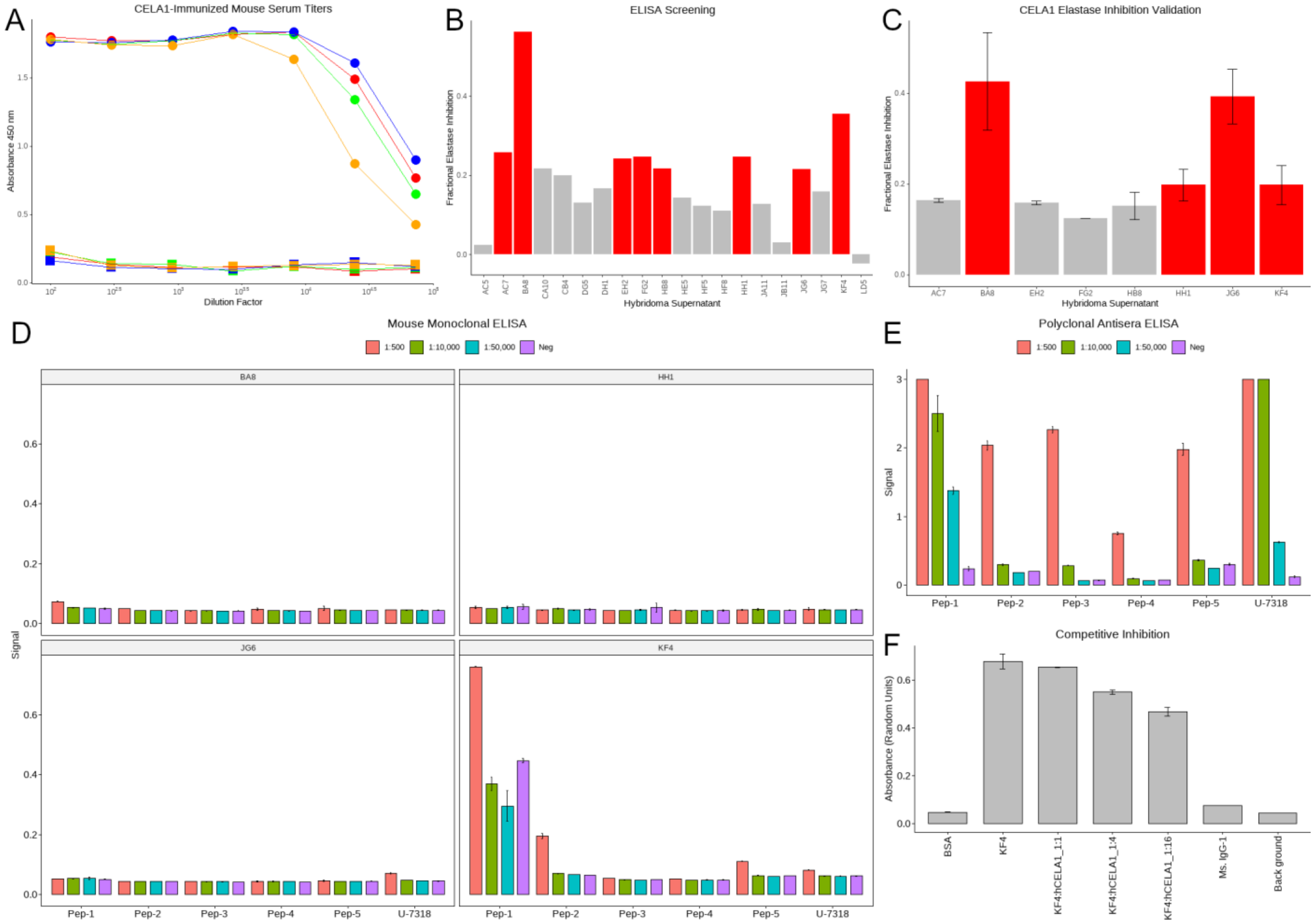

Supplemental Figure 9: Identification of KF4 as Lead Candidate. (A) The serum of four mice immunized with human CELA1 peptides all demonstrated high titers in an ELISA against recombinant CELA1. (B) Hybridomas were created and screened and the eight clones with the highest titers selected for functional screening. (C) The four clones (BA8, HH1, JG6, and KF4) with the greatest inhibition of CELA1 elastolytic activity were selected. (D) The immunizing peptides were immobilized and used for ELISA. Only KF4 detected one of the peptides used for immunization. (E) As a positive control, an anti-CELA1 polyclonal antibody was used and detected all the peptides. (F) KF4 was incubated with increasing ratios of recombinant CELA1 prior to testing by ELISA in a competitive inhibition assay. There was a serial reduction in ELISA signal.

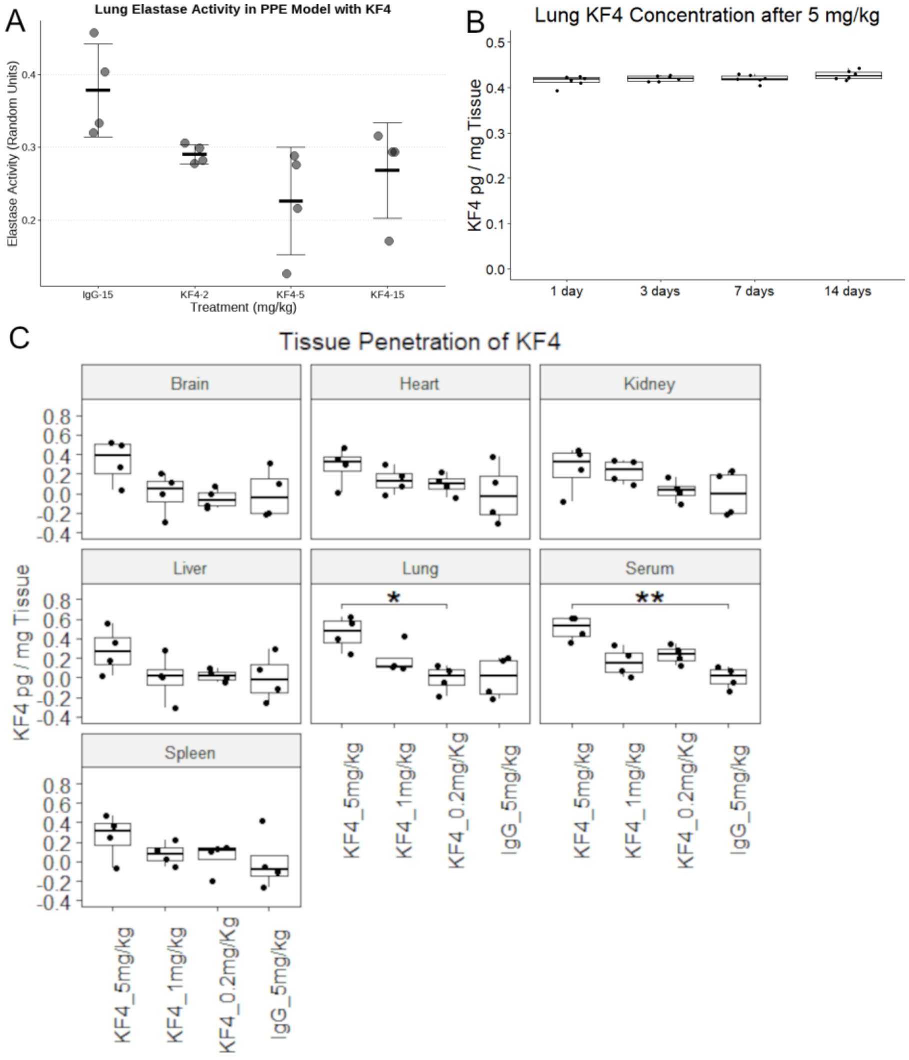

Supplemental Figure 10: KF4 Dosing and Tissue Penetration. (A) Mice were treated with PPE, and at 42 days were treated with 2, 5, or 15 mg/kg KF4 or 15 mg/kg IgG. Lung elastase activation was reduced most prominently in the 5 mg/kg dose. n=4 per group. (B) KF4 was conjugated with AlexaFlour-647 and the amount of KF4 present in lung tissue at different time points was determined. KF4 levels remained relatively constant for at least 14 days. (C) Fluorophore-conjugated KF4 was administered to mice at 0.2, 1, and 5 mg/kg. The dose dependent penetration at 24 hours in different tissues was determined by collecting, homogenizing, and quantifying the fluorescence signal of 10 mg tissue. Lung and serum Kruskal-Wallis $p < 0.05$. Dunn *post hoc* test shown. *$p<0.05$, **$p<0.01$
